# Neurotoxicity and underlying cellular changes of 21 mitochondrial respiratory chain inhibitors

**DOI:** 10.1007/s00204-020-02970-5

**Published:** 2021-01-29

**Authors:** Johannes Delp, Andrea Cediel-Ulloa, Ilinca Suciu, Petra Kranaster, Barbara MA van Vugt-Lussenburg, Vesna Munic Kos, Wanda van der Stel, Giada Carta, Susanne Hougaard Bennekou, Paul Jennings, Bob van de Water, Anna Forsby, Marcel Leist

**Affiliations:** 1 In Vitro Toxicology and Biomedicine, Department inaugurated by the Doerenkamp-Zbinden Foundation, University of Konstanz, Universitaetsstr. 10, 78464 Konstanz, Germany; 2 Cooperative Doctorate College InViTe, University of Konstanz, Konstanz, Germany; 3 Swetox, Unit for Toxicological Sciences, Karolinska Institutet, Stockholm, Sweden; 4 Konstanz Research School Chemical Biology (KoRS-CB), University of Konstanz, Konstanz, Germany; 5 BioDetection Systems BV, Amsterdam, The Netherlands; 6 Division of Drug Discovery and Safety, Leiden Academic Centre for Drug Research, Leiden University, Leiden, The Netherlands; 7 Division of Molecular and Computational Toxicology, Amsterdam Institute for Molecules, Medicines and Systems, Vrije Universiteit Amsterdam, Amsterdam, Netherlands; 8 National Food Institute, Technical University of Denmark (DTU), Lyngby, Denmark; 9 Department of Biochemistry and Biophysics, Stockholm University, Stockholm, Sweden; 10 Department of Organismal Biology, Uppsala University, Uppsala, Sweden; 11 Department of Physiology and Pharmacology, Karolinska Institutet, Stockholm, Sweden

**Keywords:** In vitro neurotoxicity, Mitotoxicity, TempO-Seq, High-content imaging, AOP:3, Mechanistic safety assessment

## Abstract

Inhibition of complex I of the mitochondrial respiratory chain (cI) by rotenone and methyl-phenylpyridinium (MPP +) leads to the degeneration of dopaminergic neurons in man and rodents. To formally describe this mechanism of toxicity, an adverse outcome pathway (AOP:3) has been developed that implies that any inhibitor of cI, or possibly of other parts of the respiratory chain, would have the potential to trigger parkinsonian motor deficits. We used here 21 pesticides, all of which are described in the literature as mitochondrial inhibitors, to study the general applicability of AOP:3 or of in vitro assays that are assessing its activation. Five cI, three complex II (cII), and five complex III (cIII) inhibitors were characterized in detail in human dopaminergic neuronal cell cultures. The NeuriTox assay, examining neurite damage in LUHMES cells, was used as in vitro proxy of the adverse outcome (AO), i.e., of dopaminergic neurodegeneration. This test provided data on whether test compounds were unspecific cytotoxicants or specifically neurotoxic, and it yielded potency data with respect to neurite degeneration. The pesticide panel was also examined in assays for the sequential key events (KE) leading to the AO, i.e., mitochondrial respiratory chain inhibition, mitochondrial dysfunction, and disturbed proteostasis. Data from KE assays were compared to the NeuriTox data (AO). The cII-inhibitory pesticides tested here did not appear to trigger the AOP:3 at all. Some of the cI/cIII inhibitors showed a consistent AOP activation response in all assays, while others did not. In general, there was a clear hierarchy of assay sensitivity: changes of gene expression (biomarker of neuronal stress) correlated well with NeuriTox data; mitochondrial failure (measured both by a mitochondrial membrane potential-sensitive dye and a respirometric assay) was about 10–260 times more sensitive than neurite damage (AO); cI/cIII activity was sometimes affected at > 1000 times lower concentrations than the neurites. These data suggest that the use of AOP:3 for hazard assessment has a number of caveats: (i) specific parkinsonian neurodegeneration cannot be easily predicted from assays of mitochondrial dysfunction; (ii) deriving a point-of-departure for risk assessment from early KE assays may overestimate toxicant potency.

**Supplementary Information:**

The online version contains supplementary material available at 10.1007/s00204-020-02970-5.

## Article highlights

- Comparison of 21 data-rich mitochondrial toxicants for neurotoxicity
- Quantitative comparison of key event triggering thresholds for AOP:3
- Comparison of two cell models and two exposure times for neurotoxicity
- Comparison of transcriptome changes and classical key event measures for sensitivity

**Supplementary Information:**

The online version contains supplementary material available at 10.1007/s00204-020-02970-5.

## Introduction

While there are many known mitochondrial toxicants, it is unclear whether they all lead to the same adverse outcomes (AO) in the nervous system. The concept of adverse outcome pathways (AOP) was established to link modifications of biological processes on a molecular/mechanistic level with an AO in humans (Leist et al. 2017). It is generally assumed that a molecular initiating event (MIE) leads to the AO by a series of key events (KE) (Allen et al. 2014). For the establishment of an AOP, the weight of evidence linking the KEs with each other needs to be assessed, quantified/rated, and documented in form of key event relationships (KER). An important feature of AOPs is that they are “chemically agnostic”, i.e., they do not describe modes-of-actions (MoA) of defined chemicals, but rather outline biological/pathophysiological processes (Bal-Price et al. 2017; Delrue et al. 2016; Vinken 2013; Zhou 2015). For example, the KE “inhibition of mitochondrial respiratory chain complex I (cI)” describes the cellular process, and not the relevant toxicant. However, each AOP definition also includes example chemicals that would trigger the respective AOP, given that they reach a sufficiently high concentration at the target of the MIE. KER are also often established on the basis of known example toxicants (Bal-Price et al. 2018; Terron et al. 2018), and most AOPs in the AOP Wiki (https://aopwiki.org/) have been established and exemplified by mining the existing literature. This means that the evidence was collected from heterogeneous sources in the scientific literature. Such studies were not designed originally to support building an AOP, and there are only very few recent examples of AOPs that are based on dedicated research to build the AOP and to parameterize KERs (Browne et al. 2015, 2017; Gijbels et al. 2020; Pistollato et al. 2020; Spinu et al. 2019).

**Fig. 1:**
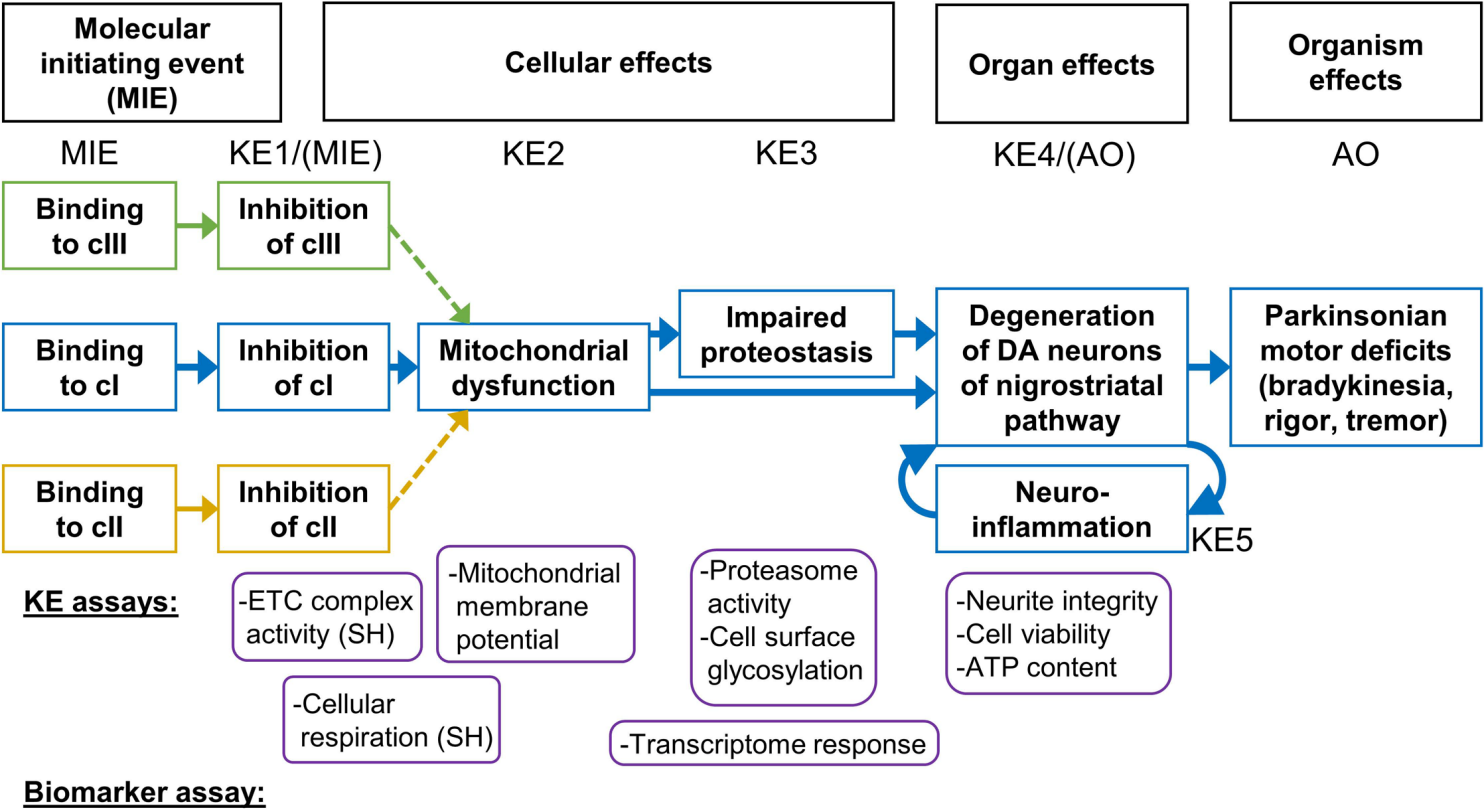
Depiction of AOP#03 with potential extensions. The adverse outcome pathway (AOP) #03 (“Inhibition of the mitochondrial complex I of nigrostriatal neurons leads to parkinsonian motor deficits”) is characterized by a sequence of key events (KE) that cause mitochondrial dysfunction and finally lead to parkinsonian motor symptoms via the selective loss of dopaminergic neurons. For mitochondrial respiratory chain complex II (cII) and cIII inhibitors, it is hypothesized that triggering their molecular initiating event (MIE) will cause mitochondrial dysfunction and might thereby trigger the same AO as cI inhibitors. Overlay of the common KE (mitochondrial dysfunction) may lead to an AOP network as displayed; modified after (Terron et al. 2018). The KE assays and biomarkers used in this study are shown below the AOP network. Note that for this particular AOP, KE1 and MIE are probed by the same assay. Moreover, KE4 qualifies as an alternative adverse outcome in classical animal studies, and it may be considered as AO for in vitro testing

The AOP:3 “Inhibition of the mitochondrial complex I of nigrostriatal neurons leads to parkinsonian motor deficits” describes how the binding to (MIE) and inhibition of mitochondrial cI (KE1) lead to mitochondrial dysfunction (KE2), impairment of proteostasis (KE3), degeneration of dopaminergic neurons in the substantia nigra (KE4), neuroinflammation (KE5), and, finally, parkinsonian motor symptoms (AO) (Fig. 1) (Bal-Price et al. 2018; Terron et al. 2018). AOP:3 is one of the few AOPs fully endorsed by the OECD, demonstrating a high level of documentation, assessment of evidence, and formalization. However, it was not the result of prospective research. Rather, the evidence was assembled post hoc. Therefore, it is not clear whether each compound capable of binding to cI would trigger AOP:3. Rotenone and MPP + , for which comprehensive studies were available, including mechanistic research and human data, have been used as main examples for AOP:3. On this basis, mechanistic plausibility could be demonstrated for epidemiological observations (Ockleford et al. 2017). Notably, the AOP:3 has particular features on both ends: i) the binding of known inhibitors to cI (MIE) always leads to inhibition of the complex (Sherer et al. 2007; Troger et al. 2020). Therefore, inhibition measurements can be used to also characterize the MIE (binding event). ii) In a regulatory context, the death of dopaminergic neurons (KE4) would be acceptable in animal studies as adverse outcome. Thus, this specific neurodegeneration can be used as an alternative AO in vitro, and it is experimentally easier accessible than the “final” AO of parkinsonian motor deficits.

The mitochondrial respiratory chain (MRC) channels electrons from nutritional substrates (e.g., carbohydrates/sugars, fatty acids or amino acids) to establish a proton gradient across the inner mitochondrial membrane (IMM). Two independent entry points for the electrons into the MRC exist: complex I (cI; NADH:ubiquinone oxidoreductase) and cII (succinate dehydrogenase; oxidation of FADH_2_). Both complexes transfer their electrons to coenzyme Q10 which delivers them to cIII (CoQH_2_-cytochrome c reductase) (van der Bliek et al. 2017). From there, the electrons flow via cytochrome c to cIV (cytochrome c oxidase), where they are finally transferred to oxygen to form water. Only complexes I, III, and IV, but not cII, translocate protons across the IMM. This proton gradient is used by cV (ATP synthase) to synthesize ATP (Rich 2003).

If assessed in animal models, mitochondrial toxicity is predicted with low sensitivity, most likely due to the fact that young and healthy animals are used, which have high compensatory capacities, so that often other adversities are observed at doses below those allowing the detection of mitochondrial toxicity. Drugs targeting the mitochondrial respiratory chain (MRC) are relatively well tolerated in normal tissues (Blecha et al. 2017). Many in vitro studies may also be insensitive to mitochondrial toxicants as cells are kept in supra-physiological (e.g., 3–5 times higher than normal plasma levels) glucose concentrations (Blomme and Will 2016; Broom et al. 2016; Perron et al. 2013; Tilmant et al. 2018).

To assess the consequences of mitochondrial impairment by potential environmental toxicants, we searched for commercially available mitochondrial cI–cIII inhibitors within the use class of pesticides (and pesticide-analogs) (Suppl. Figure 1). In the context of the European Research project EU-ToxRisk (Daneshian et al. 2016; Escher et al. 2019; Graepel et al. 2019), a set of 21 compounds was used to establish assays for mitochondrial respiratory chain functionality in neurons (Delp et al. 2019) and for testing in different non-neuronal cell systems (representing liver and kidney) (van der Stel et al. 2020). It could not be clarified in these studies, how the different modes of mitochondrial respiratory chain inhibition related to (or predicted) dopaminergic neurotoxicity, and how different assays of AOP:3 key events were correlated. To address this question, we used LUHMES and SH-SY5Y cells.

Using these models, we asked whether all KE assays gave similar inhibitor potency predictions. We hypothesized that earlier KEs may be more sensitive, and that higher concentrations were needed to trigger later KE, because more cellular mechanisms of adaptation and counter-regulation have to be overcome. This is to our knowledge the first dedicated study that used a broad set of chemicals triggering a neuronal AO and then following its effects along the AOP KEs. We addressed the important question, in how far early KE data can and will predict an AO. We assessed dopaminergic neurodegeneration (as AO), and determined the respective inhibitor potencies. From this, we worked backwards through the chain of KEs toward the KE1 (which is here overlapping with the MIE).

## Materials and methods

### Materials

Unless specified otherwise, cell culture reagents (consumables and media) were from Gibco/Thermo Fisher Scientific (Waltham, USA), and fine chemicals (inhibitors, substrates) were from Sigma-Aldrich (Steinheim, GER). CAS identifiers of the tested set of chemicals are compiled in Suppl. Fig. 1.

### Determination of cytotoxicity in U2OS cells

Cytotoxicity was determined in the Cytotox CALUX assay as follows (as also described in DB-ALM protocol 197). A cell suspension of 100,000 cells/ml was prepared in assay medium, and white 384-well plates were filled with 30 µl/well cell suspension. Twenty-four hours after seeding, a dilution series of test compound, as well as positive and negative controls, were added to the cells. After 24 h exposure, the medium was removed, and 10 µl of Triton X-100 lysis buffer was added to each well. The luminescent signal was determined in a luminometer by injecting BDS Illuminate Mix and measuring the light emission. The Cytotox CALUX cell line constitutively expresses a high level of luciferase protein; the light emission is therefore proportional to the cell number of live cells, and its decrease can be used as measure of cytotoxicity. The light emission generated by cells exposed to vehicle control was set to 100%, and the luminescence of cells exposed to the positive control toxicant tributyltin acetate [100 µM] was set to 0%. The light output of the cells exposed to different concentrations of test compounds was scaled accordingly.

### SH-SY5Y cell culture and differentiation

SH-SY5Y cells originate from the bone marrow biopsy of a young neuroblastoma case (ATCC CRL-2266). The cells are able to form neurites and display several dopaminergic characteristics. For this reason, they are frequently used in neuropathology and neurotoxicity research (Cheung et al. 2009; Forsby et al. 2009; Gustafsson et al. 2010; Lopes et al. 2010; Nordin-Andersson et al. 2003; Okuda et al. 2006; Xie et al. 2010). Cells were plated in 100 µl proliferation medium (EMEM supplemented with 10% fetal bovine serum, 2 mM L-glutamine, 1% non-essential amino acids, 100 µg/ml streptomycin and 100 U/ml penicillin) at 37 ℃ in a 5% CO_2_/95% air atmosphere (Gustafsson et al. 2010). Differentiation was initiated 24 h later (on day 0, d0) by exchange to 100 µl/well differentiation medium (DMEM:F12, supplemented with 1 µM all-trans retinoic acid (RA), 1 × N2 supplement, 2 mM l-glutamine, 100 µg/ml streptomycin, and 100 U/ml penicillin). All media and supplements were obtained from Gibco (ThermoFisher) except from RA (Sigma).

### Neurite degeneration and cytotoxicity assessment using SH-SY5Y cells

Cells were plated in black 96-well CELLSTAR^®^ plates with micro-clear bottom (Greiner) at a density of 23,500 cells/cm^2^ and differentiated as described above. On d3, 100 µl of fresh differentiation medium were added to the wells. For the 120 h exposure, this medium contained twice the final treatment concentration. On d6, 100 µl from each well were removed, and the procedure was repeated and the cells were analyzed on d8. For the 24 h exposure, cells received an addition of 100 µl pure differentiation medium on d3 and were treated with compounds as described above on d6 for 24 h before analysis. After exposure to the chemicals, 100 µl per well was removed and 20 µl of a stain mixture [6 µg/ml calcein-AM, 60 µg/ml Hoechst 33,342, and 6 µM propidium iodide (PI; only for 120 h exposures) in DMEM:F12] was added to each well to visualize cells and neurites, nuclei and dead cells, respectively. The cells were incubated with the stain mixture for 20 min at 37 °C, 5% CO_2_. After incubation, 130 µL of differentiation medium were added to all the wells to stop further staining of the cells. Images were obtained using ImageXpress^®^ Micro (Molecular Devices). The cells were imaged with a 10 × objective lens; the fluorescence from the calcein stained cell bodies and neurites was detected in the FITC channel (excitation 475 ± 34 nm, emission 536 ± 40 nm). Nuclei were identified on the DAPI channel (excitation 377 ± 50 nm, emission 447 ± 60 nm) and the PI positive cells on the Texas red channel (excitation 562 ± 40 nm, emission 624 ± 40 nm). The mean neurite length was calculated using the Neurite Outgrowth plug-in in the MetaXpress® Software. Outgrowths were defined as cytoplasmic prolongations with a maximum width of 2 µm (3 pixels). The minimum outgrowth size needed to be logged as significant was defined as 15 µm (23 pixels). Nuclear staining with Hoechst 33342 and PI was determined using the transfluor HT plug-in in the MetaXpress® Software. Cell viability was determined by the ratio of viable cells (PI—negative) relative to the overall cell count (Hoechst 33342—positive).

### LUHMES cell culture

LUHMES cells are conditionally immortalized human neuronal precursors, which can be differentiated into dopaminergic neurons (Gutbier et al. 2018a; Scholz et al. 2011). They have been characterized extensively with regards to their sensitivity to the parkinsonian toxicant MPP + /MPTP (Harischandra et al. 2020; Pierce et al. 2018; Schildknecht et al. 2009, 2015) as well as their metabolism and transcriptome changers triggered by MPP + (Delp et al. 2018a; Krug et al. 2013b, 2014). The cells have also been used for research on neurodevelopmental disorders (Krug et al. 2013a; Matelski et al. 2020; Stiegler et al. 2011), as well as for high-throughput toxicity screenings and neuron–glia interactions (Brüll et al. 2020; Delp et al. 2018b; Efremova et al. 2015; Gutbier et al. 2018b; Tong et al. 2017; Witt et al. 2017). Cells were cultured as described earlier (Scholz et al. 2011). In brief, the proliferation culture was maintained in PM [i.e., Adv. DMEM/F12 supplemented with 2 mM l-glutamine, 1 × N2 supplement (Invitrogen), and 40 ng/ml recombinant human basic fibroblast growth factor (R + D systems)] at 37 °C in a humidified 5% CO_2_/95% air atmosphere. Cells were passaged every other day using 0.05% trypsin/EDTA (Invitrogen). Differentiation was initiated by changing the medium to DM [Adv. DMEM/F12 supplemented with 2 mM l-glutamine, 1 × N2 supplement (Invitrogen) and 1 mM N6,2′-O-dibutyryl 3′,5′-cyclic adenosine monophosphate (cAMP) (Sigma-Aldrich), 1 µg/ml tetracycline (Sigma-Aldrich), and 2 ng/ml recombinant human glial cell-derived neurotrophic factor (GDNF, R & D Systems)]. Culture plastic ware (Sarstedt) was pre-coated with 50 μg/ml poly-l-ornithine (PLO) and 1 μg/ml fibronectin (Sigma-Aldrich).

### LUHMES differentiation and exposure schemes (i.e., NeuriTox scheme)

The NeuriTox test was performed as previously described (Delp et al. 2018b; Krug et al. 2013a; Stiegler et al. 2011). Briefly, cells differentiated for 48 h were re-seeded into 96-well plates at a density of 100,000 cells/cm^2^ in DM without cAMP and GDNF, and treated 1 h after seeding for 24 h. Subsequently, multiple endpoints were determined, e.g., neurite outgrowth (i.e., NeuriTox test), resazurin reduction, intracellular ATP content, gene expression levels from TempO-Seq analysis, and proteasomal activity. For the 120 h exposure scenario, LUHMES cells were differentiated for another 3 days after reseeding before they were treated on day 5 (d5) and d7 by a half medium exchange. Endpoints were determined on d10 of differentiation.

### LUHMES/NeuriTox test image acquisition and analysis

Image acquisition and analysis was performed as described previously (Krug et al. 2013a; Schildknecht et al. 2013; Stiegler et al. 2011). In brief, cells were live-stained with 1 µg/ml Hoechst H-33342 and 1 µM calcein-AM and image acquisition was done by an automated high content imager (Cellomics, Waltham, MA, USA). Cell viability was determined as ratio of live cells (cell bodies being Calcein- and Hoechst-positive) relative to the total amount of cells (Hoechst-positive cell bodies). Neurite area was calculated by subtracting the somatic area (Hoechst-positive area plus surrounding) from the calcein-positive area.

### Determination of the intracellular ATP content and resazurin reduction

Plates from the NeuriTox test were further used after imaging for determination of alternative endpoints. Intracellular ATP was determined as described earlier (Delp et al. 2019). In brief, CellTiterGlo 2.0 assay mix (Promega) containing luciferase was added after imaging, and luminescence was recorded. For the determination of resazurin reduction, a 10 × resazurin stock solution was added after imaging to the medium (final resazurin concentration 2 µg/ml) (Delp et al. 2018a). After 60 min incubation, resazurin fluorescence was recorded (ex: 530 nm, em: 590 nm). Background-corrected data was used for further analysis.

### Proteasomal activity (KE3 assay)

Activity of the proteasome was quantified as described in (Gutbier et al. 2018b). Briefly, after exposure, culture medium was replaced by assay buffer containing a cell-permeable proteasomal substrate (MeOSuc-Gly-Leu-Phe-AMC, 25 µM (Bachem, Bubendorf, Switzerland) in HBSS). Its AMC-fluorescence (ex: 360 nm, em: 465 nm) increases with proteasomal activity and was monitored for 90 min. Background-corrected data were used for further analysis.

### *Detection of proteostasis impairment *via* NeuroGlycoTest (KE3 assay)*

The NeuroGlycoTest was performed as previously described (Kranaster et al. 2020). In brief, d6 LUHMES cells were co-treated for 6 h with the respiratory chain inhibitors together with 10 µM azide-tagged mannosamine precursor sugar (Ac4ManNAz), synthesized according to published procedures (Saxon et al. 2002). After PBS-washing, a dibenzylcyclooctyne-PEG_4_-biotin (DBCO-biotin) [100 μM] (Jena Bioscience, Jena, Germany) solution was added for 20 min at 37 °C to label the incorporated azide-tagged sialic acids (= SiaAz).

For detection of MGE-sialoproteins, cells in 6-well plates were lyzed in Laemmli buffer and boiled for 5 min at 95 °C. After SDS-PAGE and Western blotting, membranes were probed with a goat HRP-conjugated anti-biotin antibody (Cell Signaling Technology, Massachusetts, US), with a mouse glyceraldehyde 3-phosphate dehydrogenase (GAPDH) monoclonal antibody ZG003 (Thermo Fisher Scientific, Massachusetts, US), and with a goat anti-mouse IgG horseradish peroxidase-conjugated secondary antibody. Relative band intensity was quantified using a self-developed image evaluation software. Intensities were normalized to corresponding GAPDH loading control. At least two blots from independent experiments were quantified.

For detection of neurite MGE, cells in 8-well µ-slides were further incubated with a mixture of streptavidin-Alexa Fluor 488 [8 μg/ml] (to fluorescently label the biotin-tagged sialic acids), CellTrace™ Calcein Red–Orange [10 μM] (Thermo Fisher Scientific, Waltham, Massachusetts, US) (to visualize cellular cytoplasm), and H-33342 [1 µg/ml] (to label nuclei) for 20 min at 37 °C in the dark. After PBS-washing, cells were fixed with 2% paraformaldehyde containing 4% sucrose. Microscopy was performed using a Zeiss LSM 880 point laser scanning confocal microscope. Five images were taken per condition as z-stack (total z-range of 4 µm), and processed using Fiji. The processed images were analyzed using the SUIKER software (Karreman et al. 2019) that simultaneously, from the same image, detected and quantified viability (based on the Hoechst staining), neurite area (based on the Calcein staining) and neurite MGE sialic acids (based on the streptavidin-Alexa Fluor 488 label). Detailed descriptions of the image analysis as well as the software are freely accessible at https://invitrotox.uni-konstanz.de/Suiker/.

### Determination of mitochondrial membrane potential and resazurin reduction in SH-SY5Y cells (KE2 assay)

Cells were plated at a cell density of 29,500 cells/cm^2^ and handled as described above for the neurite SH-SY5Y degeneration. On d6 of differentiation, 50% of the medium was replaced by differentiation medium containing 2 µM Rhodamine 123 (ThermoFisher). After 45 min of preloading at 37 °C with 5% CO_2_ in darkness, the Rhodamine 123 solution was completely removed and cells were carefully washed once with 200 µl DMEM:F12. Subsequently, 50 µl differentiation medium per well were added followed by the addition of 50 µl/well of test compounds in medium. After 24 h of exposure to the test compounds, the nuclei were stained for 30 min with 20 µL of a solution containing 20 µM Hoechst 33342, and the images were then acquired with a 10 × objective lens using the ImageXpress® Micro (Molecular Devices) high content microplate imager. Nuclei were evaluated in the DAPI channel (excitation 377 ± 50 nm, emission 447 ± 60 nm), whereas Rhodamine 123 fluorescence was detected in the FITC channel (excitation 475 ± 34 nm, emission 536 ± 40 nm). Analysis of the Hoechst 33,342 and Rhodamine 123 fluorescence was performed using the MetaXpress^®^ Software (Molecular Devices) with the Transfluor HT plug-in and the parameters nuclei (for Hoechst-33342) and integrated granule intensity (for Rhodamine 123). Mitochondrial membrane potential (MMP) was quantified as the total fluorescence intensity of Rhodamine 123 in relation to the number of Hoechst-stained nuclei.

For the quantification of resazurin reduction, unstained cells were treated on d6 as described above. After 24 h exposure, 150 µl of the toxicant solution was removed from each well and 50 µl of 88 µM resazurin in DMEM:F12 medium were added (final concentration 44 µM). After 120 min incubation, the fluorescence intensity of reduced resazurin was recorded using a Tecan reader (ex 540 ± 9 nm, em 590 ± 20 nm).

### Assessment of mitochondrial respiration of intact cells (KE1/2 assay)

Respiration of intact LUHMES cells was performed as described earlier (Delp et al. 2018a, 2019). Briefly, 48 h differentiated cells were re-seeded into Agilent Seahorse XFe24-well plates in DM without cAMP and GDNF at a density of 100,000 cells/cm^2^ and cultured for 24 h before the assay was initiated. Inhibition of respiration was calculated as the difference between baseline measurements (i.e., after equilibration, last measurement before treatment) and the first measurement after treatment with the test compound. To analyze the impact on mitochondrial respiration only, non-mitochondrial respiration (retrieved by subsequent treatment with 1 µM rotenone and 0.5 µM antimycin A) was subtracted from all values before analysis.

### Quantification of individual respiratory chain complexes using permeabilized LUHMES cells (MIE/KE1 assay)

To investigate individual respiratory chain complexes, a method developed earlier and described extensively was used (Delp et al. 2019). In brief, LUHMES cells were seeded at a density of 205,000 cells/cm^2^ in PM into Agilent Seahorse XFe24-well plates. After culture for 24 h, cells were permeabilized by replacing the medium with MAS buffer [(220 mM mannitol, 1 mM ADP, 70 mM sucrose, 10 mM KH_2_PO_4_, 5 mM MgCl_2_, 2 mM HEPES, 1 mM EGTA, 4 mg/ml fatty acid free BSA, and pH = 7.2] supplemented with 25 µg/ml digitonin. Thirty minutes after permeabilization and equilibration, Seahorse measurements (oxygen consumption rate, OCR) were initiated. Complex-specific substrates were used to drive specific respiration, while inhibitors were used to block the electron influx into the ETC from earlier complexes [cI: substrate was 5 mM Pyr, 2 mM Gln, and 2.5 mM Mal, inhibitor was 0.5 µM rotenone; cII: substrate was 10 mM succinate, inhibitor was 5 mM malonate; cIII: substrate was 250 µM duroquinol (TCI chemicals Germany, Eschborn, GER); inhibitor was 0.5 µM antimycin A; cIV: 125 µM TMPD + 2 mM ascorbic acid, no specific inhibitor was used]. To reduce data variability, internal normalization of the OCR of each well was done relative to its value after equilibration.

### Generation and processing of LUHMES TempO-Seq data

Cells were cultured and treated as described above for the NeuriTox assay. On d3, after 24 h treatment, culture medium was replaced by 33 µl/well 1 × Biospyder lysis buffer. After 15 min at room temperature, plates were sealed and frozen at -80 °C to complete lysis. Samples were analyzed at Bioclavis (Glasgow) using the TempO-Seq technique and the EU-ToxRisk v2.0 gene probe panel covering ca. 3000 genes (Yeakley et al. 2017). All genes are listed in the supplementary excel file (containing also all gene regulations). Briefly, a 50 bp fragment of the target gene was amplified. The amplification step also introduced sample-specific barcodes. This allowed pooling of all samples and standard next-generation sequencing of the ensemble. Afterward, adapters were used to allocate the sequenced fragments to the correct samples. Finally, the alignment of all sequenced fragments (FASTQ files) for each sample to the collection of reference 50 bp oligonucleotides was performed for the quantification of reads per gene, also known as the read count values. In a pre-filtering step, samples with a total count < 0.2 million and genes with an average count < 1.5 were removed. The effect of normalization was checked by boxplots and distribution plots (not shown), and no outlier samples were identified.

To correct for both read depth and RNA composition, the DESeq2′s median of ratios normalization was applied. The analysis of differentially expressed genes (DEG) of each treatment against the control group was done by the Wald test implemented in DESeq2/R (Love et al. 2014), including an FDR correction for p-adjusted ≤ 0.05. To retrieve the effective concentration that caused 10% DEGs (EC_10_DEG), the total (upregulated + downregulated) number of DEGs per treatment condition was analyzed. A curve was fit through these concentration–response data to obtain EC_10_DEG. For comparability, all DEG data were normalized to a positive control. We used 13 µM deguelin (i.e., the EC_10_V of deguelin) as positive control, and it triggered 325 DEGs. This response was set as 100% and all other DEGs’ numbers were expressed relative to it; the response was limited to be no higher than 100%, i.e., DEG numbers between 0 and 325 resulted in 0–100% response, and DEG numbers > 325 were all set to full response (= 100%). The EC_10_DEG was calculated using an established online open access BMC calculator (BMCeasy) (Krebs et al. 2020).

### Curve fitting, data mining, and statistics

If not mentioned otherwise, at least three independent experiments (= biological replicates, run from different cell batches) were performed. Data of treated samples were expressed relative to DMSO solvent controls, and they are reported as means ± SEM. To calculate curve fits, a 4-parameter Hill model was used and the upper and lower asymptotes fixed to 100 and 0%. Effective concentrations (EC) were determined by solving the Hill equation for *x*%, e.g., 10% (for *x* = 10) reduction of viability for the EC_10_V. Analysis of statistical differences was specified at individual methods sections (e.g., gene expression data) or figure legends (e.g., Figs. 1 and 5). For concentration–response data that were meant to give general overviews of effects and of the ranges of effect sizes, statistics were not performed for individual data points. This has several reasons. The two most import ones are that (i) in most cases, not the data points as such, but a summary measure of the curve, e.g., BMC_10_, was the main research result, and second (ii) for the multiple comparisons within and across many dimensions, most standard statistical approaches would yield problematic results or suggest an inappropriate exactness, given that data are interdependent to a certain degree, and that low *n*-numbers do not allow a solid basis for simple key assumptions (like normal distribution, etc.) when so many (hundreds of) comparisons are to be performed.

## Results and discussion

### Assessment of key event activation along AOP#03 and potential extensions of it

The adverse outcome pathway (AOP) #3 was used to guide the experiments performed in this study on inhibitors of mitochondrial respiratory chain (MRC) complexes I, II, and III (cI, cII, cIII). AOP:3 describes the binding (MIE) and inhibition (KE1) of a substance to cI, which subsequently leads to mitochondrial dysfunction (KE2), impaired proteostasis (KE3), nigrostriatal dopaminergic degeneration (KE4), and neuroinflammation (KE5). This may culminate in parkinsonian motor symptoms (AO) (Fig. 1) (Terron et al. 2018). As cII and cIII inhibitors are also expected to impair mitochondrial function, we investigated whether an extended AOP:3, starting at cII or cIII, could be substantiated with data indicating a link between cII/cIII inhibition and dopaminergic neurodegeneration, or whether cI inhibition is a special situation that is not recapitulated by other MRC inhibitors. To assess this, KE and biomarker assays were utilized to evaluate the activation of events downstream of the AO.

### Application of the NeuriTox test as KE4 assay to identify dopaminergic neurotoxicants (AO).

**Fig. 2:**
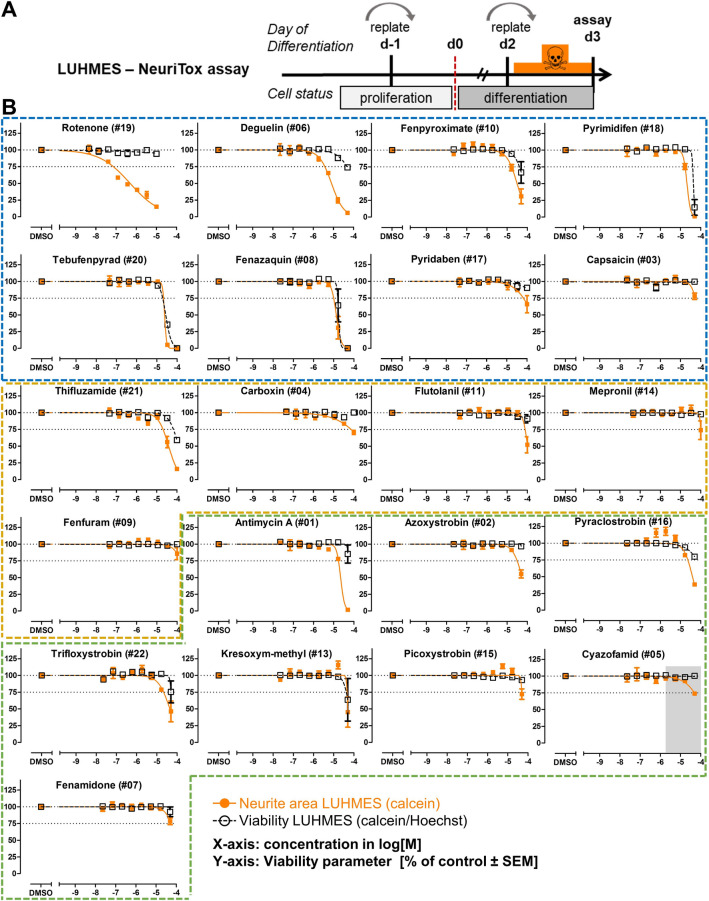
Application of the NeuriTox test as KE4 assay to identify dopaminergic neurotoxicants (AO). **a** LUHMES cells were differentiated for 48 h, and treated for 24 h after replating during their differentiation phase. Neurite outgrowth (NA, orange) and viability (V, black) were assessed on day 3 (d3) of differentiation by automated high-content imaging with calcein/H-33342 staining. **b** Graphs for individual compounds were ordered according to the described mode of action of the test compounds (cI, II, III inhibitors in blue, yellow, and green boxes) and their potency within their group. Gray area: compound was insoluble in that concentration range. Data are means ± SEM from three independent experiments. The NeuriTox assay has an established prediction model, in which reductions of the neurite area by ≥ 25% are considered as (positive) hit (Delp et al. 2018a, b). Therefore, significance was not tested for individual data points, but the threshold is indicated by dotted lines (color figure online)

To directly assess which of the cI–cIII inhibitors might be specific neurotoxicants causing dopaminergic neurodegeneration, the LUHMES-based NeuriTox assay was used as in vitro proxy (of in vivo degeneration and motor dysfunction). The test relies on the simultaneous assessment of neurite outgrowth and general cell viability (Fig. 2a) (Krug et al. 2013a; Stiegler et al. 2011). A strong heterogeneity regarding the compounds’ potency and specificity (i.e., neurite damage without decrease in viability) was observed (Fig. 2b). Only rotenone and deguelin were active in the sub-µM range, and only rotenone and deguelin were specifically neurotoxic [i.e., EC_25_(V)/EC_25_(NA) > 4]. For some compounds, the viability did not decrease sufficiently to allow calculation of the EC_25_(V)/EC_25_(NA) ratio. They might also be specifically neurotoxic, but at higher concentrations. For many substances, especially cII and cIII inhibitors, no neurite effects > 25% were detected (definitely no specific neurotoxicity in the tested concentration range).

### Assessment of neurite degeneration in SH-SY5Y cells after either 24 or 120 h exposure

**Fig. 3:**
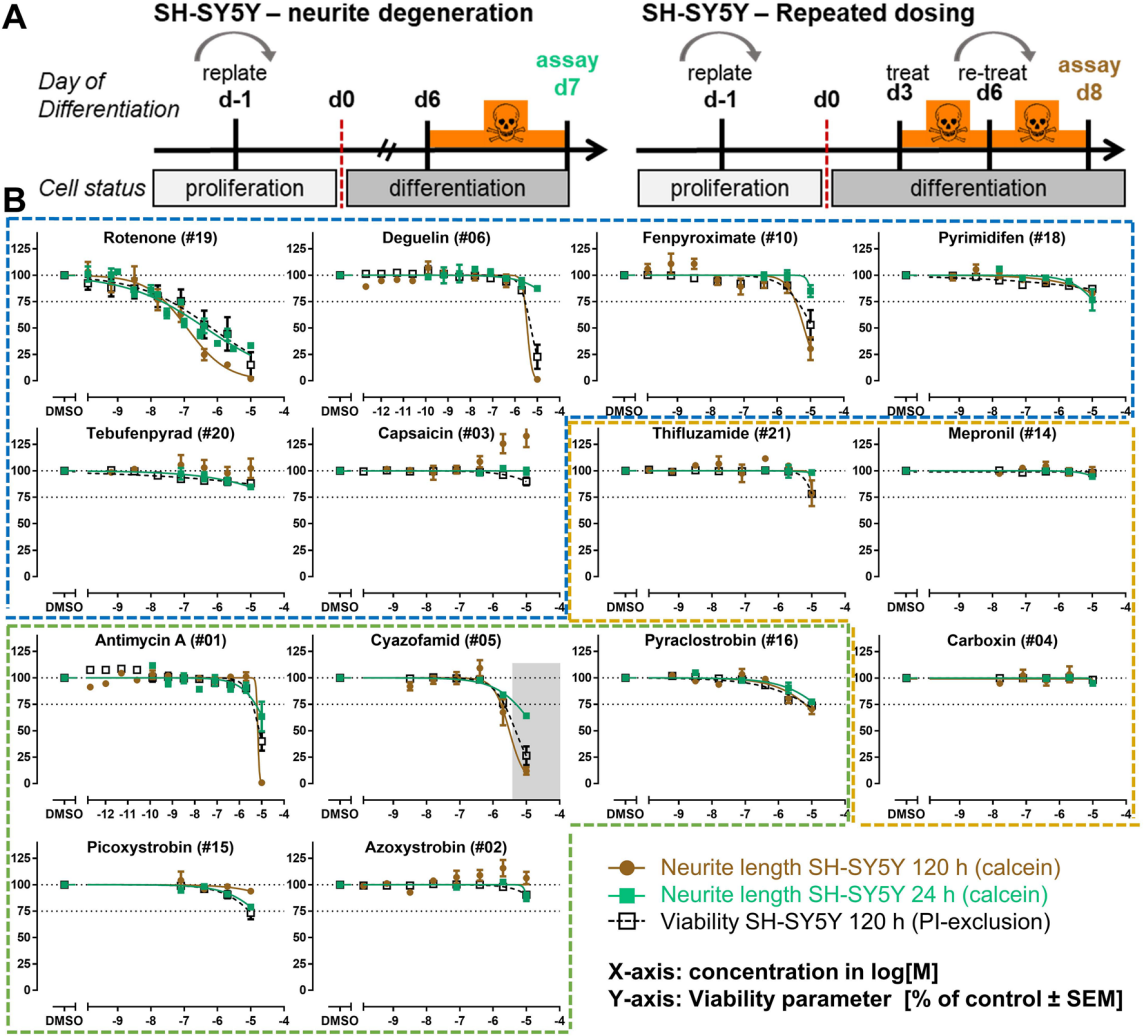
Assessment of neurite degeneration in SH-SY5Y cells after either 24 or 120 h exposure. **a** For the short exposure scenario (left), SH-SY5Y cells were differentiated for 6 days and treated once for 24 h (green). For the long-exposure scenario (right), the cells were differentiated for 3 days, subsequently treated 3 days, and retreated for another 2 days (brown). Neurite length and viability (black) were determined on day 7 (24 h exposure) or day 8 (120 h exposure), using high-content imaging and calcein/propidium iodide staining. **b** Concentration–response graphs for cI (blue), cII (yellow), and cIII (green) inhibitors, ordered according to the compounds’ potency within their MoA group. Note: only the 120 h viability data were plotted, since a 24 h exposure did not affect viability under any condition tested. Data are means ± SEM from three independent experiments (color figure online)

As secondary, independent confirmation of the NeuriTox results, neurite degeneration was observed in two SH-SY5Y-based assays, after either 24 h or 120 h treatment (Fig. 3a). Viability was not affected for any of the compounds after 24 h, at concentrations up to 10 µM (not shown). Even after 120 h exposure, only a few compounds (rotenone, deguelin, fenpyroximate, antimycin A, and cyazofamid) caused a reduction in viability by > 25% and a degeneration of neurites after exposure with up to 10 µM inhibitor (Fig. 3b). Three out of six cI inhibitors and two of five cIII inhibitors caused neurite effects > 25%. In summary, the heterogeneous results from the NeuriTox test were largely recapitulated in SH-SY5Y cells.

Similar to the NeuriTox test, rotenone and antimycin A caused a specific neurite degeneration > 25% after 24 h, without affecting viability. Cyazofamid also induced > 25% neurite degeneration without any signs of cytotoxicity in the SH-SY5Y cells, but no effects were observed after 24 h exposure with up to 10 µM deguelin. Effects on neurite degeneration were largely similar between 24 and 120 h exposure scenarios (Suppl. Fig. 3C). However, 120 h exposure increased neurite degeneration, but also cytotoxicity was observed for deguelin, fenpyroximate, and cyazofamid at the same concentrations. This could be due to intracellular accumulation of the substances upon longer/repeated exposure.

In SH-SY5Y cells, 24 and 120 h exposure data were in concordance for ten of 14 investigated compounds. A similar comparison was done in LUHMES cells, where also a 120 h exposure scenario was compared to the standard 24 h exposure (Suppl. Fig. 3a). For all compounds, neurite effects were in a similar range (less than tenfold difference) for 24 and 120 h exposures (Suppl. Fig. 3b), but the EC_25_ values were in most cases slightly lower for the 120 h data set (Suppl. Fig. 3c). For pyrimidifen, we observed a 9.8-fold increase in sensitivity. For deguelin, thifluzamide, fenpyroximate, pyraclostrobin, and cyazofamid, the increase was five-to-eightfold. Neurite degeneration for all these compounds was also enhanced to a similar degree in SH-SY5Y cells after prolonged exposure (Fig. 3). However, longer exposures also had the disadvantage that viability decreased stronger after 120 h than after 24 h, triggering the problem that discrimination between specific neurotoxicants and general toxicants becomes difficult.

### Assessment of specific neurotoxicity

**Fig. 4:**
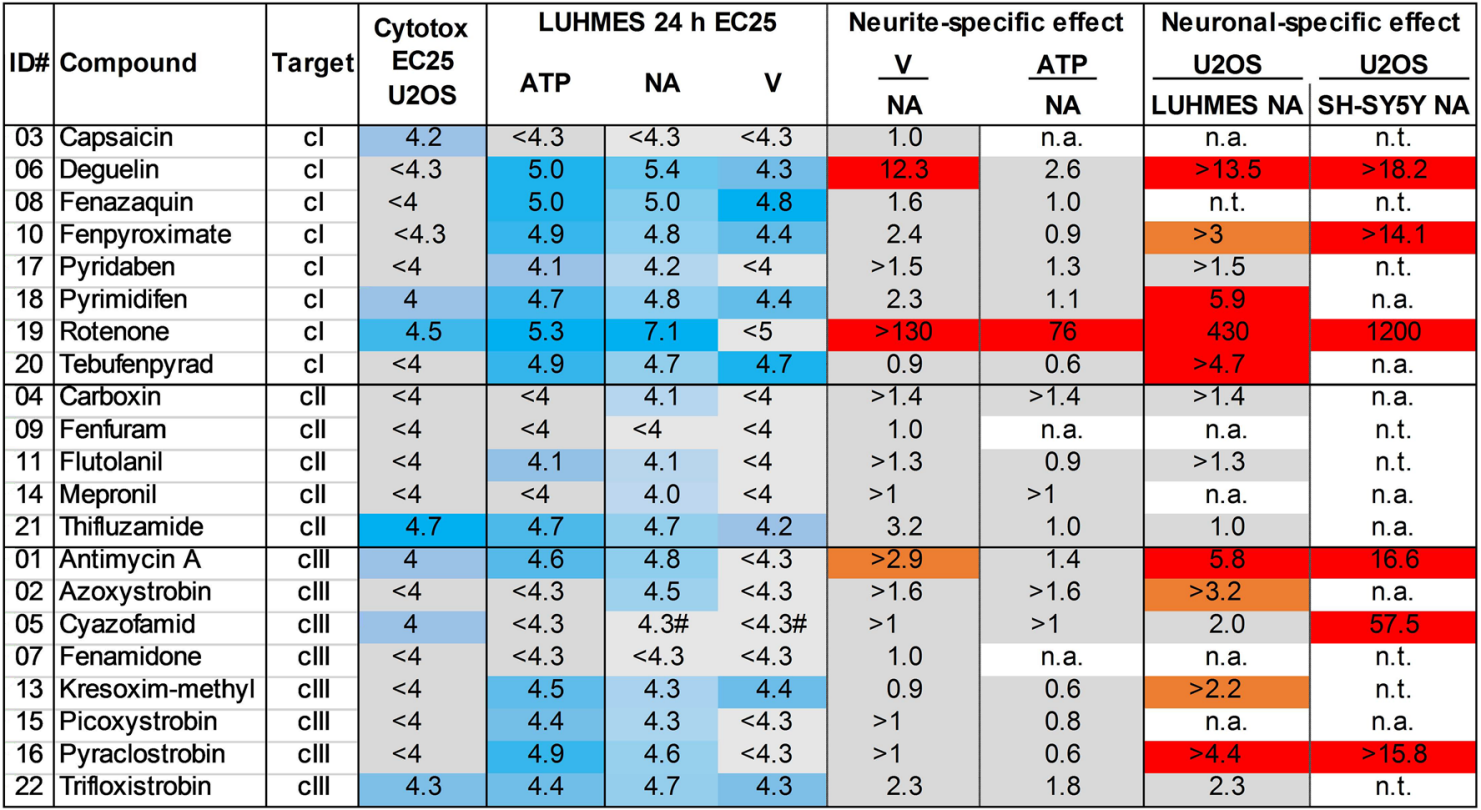
Synoptic overview of measures of specific neurotoxicity. Compounds are grouped according to their MoA. Columns 4–7 of the table indicate the potency of the test compounds for different test endpoints, are given in − log[M], and refer to the EC25 values. Concentrations were coded from intense blue (= highly active, low concentration) to light blue (= less active, high concentration); when the highest tested concentration did not result in 25% effect, the cell was colored gray. Columns 8–11 give potency ratios, calculated using potency data in their non-logarithmic form. Ratios were colored according to the established prediction model of the NeuriTox test, i.e., red if > 4 (= specific neurotoxicity), gray if < 4, i.e., unspecific, and orange if the ratio could not be fully calculated due to a lack of effect in the assessed concentrations, but was ≥ 2. Unspecific cytotoxicity was assessed using U2OS cells (osteosarcoma; treated for 24 h, cytotoxicity was measured by luminescence of constitutively expressed luciferase). The LUHMES 24 h endpoints were generated following the NeuriTox exposure scheme as in Fig. 2; the SH-SY5Y endpoint was determined by treating the cells for 120 h (data taken from Fig. 3). cI-III: electron transport chain complex I, II, or III; V: viability; NA: neurite area, n.t.: not tested; n.a.: ratio could not be calculated as compound did not trigger toxicity; #: cyazofamid was found to precipitate in the active concentration range; thus, it was considered to be inactive (color figure online)

We also pursued another concept to define specific neurotoxicity: cytotoxicity data from LUHMES and SH-SY5Y were compared to data from an unrelated, non-neuronal cell line, i.e., U2OS carcinoma cells (Fig. 4). The neurite area of LUHMES cells (or neurite length in SH-SY-5Y cells) was on average more sensitive to MRC inhibitors than U2OS viability. However, the observed effects were heterogeneous within cI inhibitors, with rotenone showing a > 100-fold offset, deguelin a > tenfold offset and other compounds rather with one-to-fivefold offsets. For cIII inhibitors, also large difference were observed (antimycin A being a potentially strong neurite-specific toxicant). It was also of interest to correlate effects on intracellular ATP levels with viability effects. Most compounds decreased ATP at similar concentrations as they affected the neurites. A notable exception was again rotenone. This extensive comparison showed that compounds with apparently similar modes of action (MRC inhibition) may show distinct toxicological effects. Both neuronal cell models showed a high concordance in the prediction of potentially specific neurotoxicity, whatever approach was applied. Notably, all data suggested that cII inhibitors have a low neurotoxic potency.

### Assessment of proteostasis endpoints for KE3

**Fig. 5:**
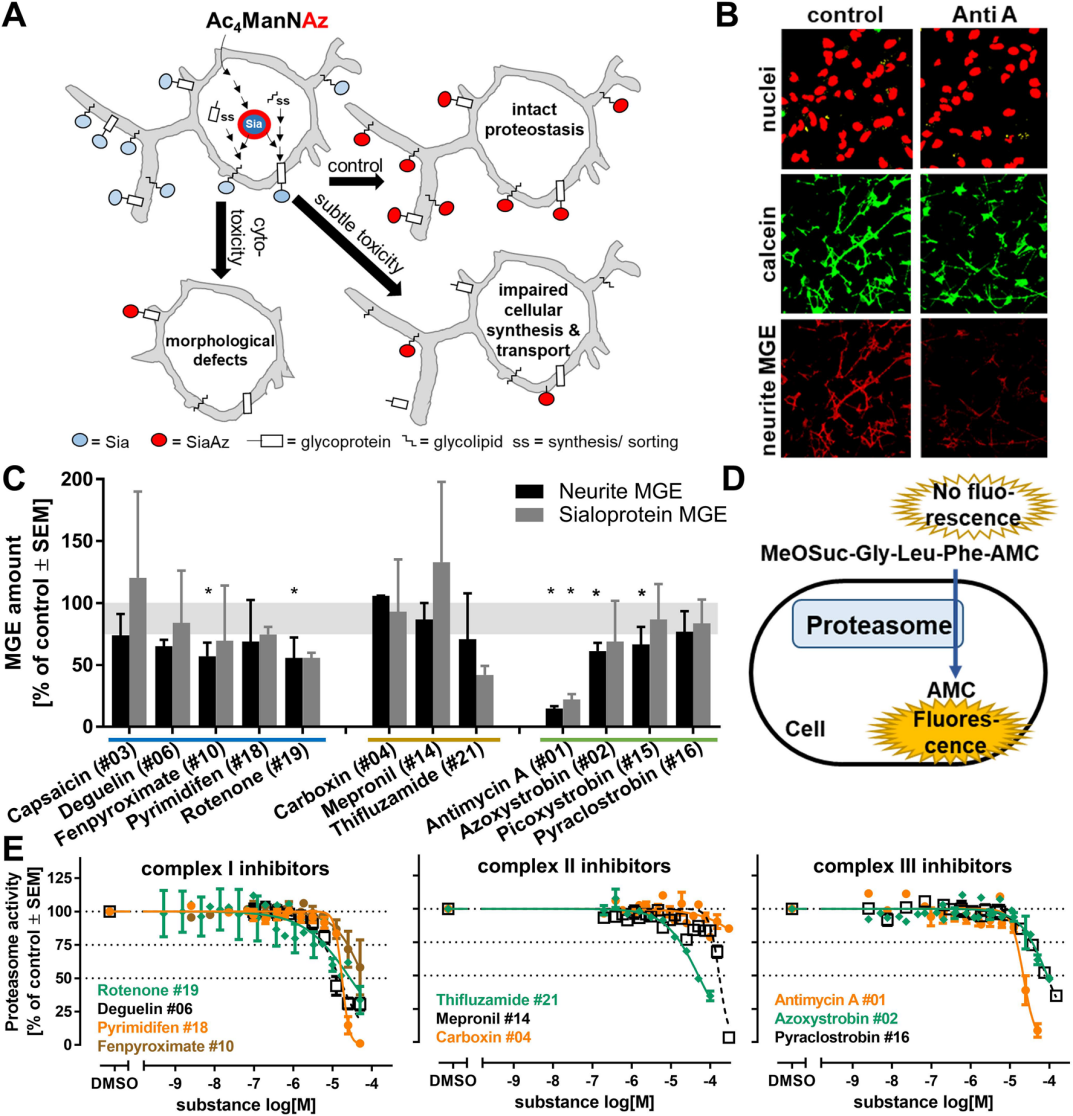
Assessment of proteostasis endpoints. **a** Proteostasis consists of several processes, including synthesis, sorting, and glycosylation of lipids and proteins. These are assessed by the NeuroGlycoTest, which is based on metabolic glycoengineering (MGE), i.e., feeding of modified mannosamine sugars (peracetylated N-azidoacetylmannosamine, Ac4ManNAz) to cells. These are used instead of their natural analogs (N-acetylmannosamine, ManNAc) by glycosylation enzymes. These sugars become covalently linked to the proteins and lipids (as azide-modified sialic acid: SiaAz/Sia), and are then transported to the cell membrane. If toxic compounds interfere with any step of this proteostatic process, a reduced cell surface glycosylation can be detected (modified from (Kranaster et al. 2020)). **b** Example pictures of LUHMES cells treated for 6 h with 50 µM antimycin A (Anti A) or vehicle control. The first row shows nuclei stained with H-33342 as control for cell number and viability. The second row shows neurites stained with calcein (cell bodies were removed by image analysis algorithm). The third row displays the labeled sialic acids (as stained by MGE and a subsequent color reaction) on neurites. Pictures have a width of 166 µm. **c** A subset of 14 test compounds was investigated at their highest non-toxic concentration (concentrations given in Suppl. Figure 4) for effects on the sialic acid content on the surface of neurites (neurite MGE). As second endpoint, cells were lysed and the total amount of MGE labeled sialoproteins was determined by Western blot (sialoprotein MGE). Both endpoints were normalized to DMSO controls of two independent experiments. Error bards indicate the data range. To identify significant changes, a one-way ANOVA followed by Fisher’s LSD test was performed, *: *p* < 0.05. **d** To investigate the proteasomal activity as alternative indicator of proteostasis, d2 LUHMES cells were incubated with toxicants for 22 h. Subsequently, cell culture medium was replaced by assay buffer containing a cell-permeable proteasomal substrate. The coumarin fluorescence (AMC) generated by the proteasomal activity was quantified as described earlier (Gutbier et al. 2018b). **e** The proteasome activity was determined for selected cI-III inhibitors. Data are means ± SEM from three to four independent experiments

After this broad coverage of KE4/AO by various in vitro assays, we worked our way backwards along the AOP. KE3 (disturbed proteostasis) was examined next. Proteostasis is a complex process, and we chose an assay that includes several cellular functions (comprising synthesis and sorting of proteins and lipids). In simple terms, the metabolic glycoengineering (MGE) assay measures the efficiency of cell surface glycolipid and glycoprotein production from a sugar precursor (Fig. 5a). For this purpose, a labeled sugar (precursor) was fed to LUHMES cells, and the display of this sugar (= sialic acid) on the cell surface in the form of glycolipids and glycoproteins was quantified by a fluorescent staining and imaging method (Kranaster et al. 2020). As exemplified for antimycin A, a strong decrease in the neurite MGE signal (glycolipids/glycoproteins on the surface of neurites) was observed in the absence of cell death. The toxicant even led to a reduction of the MGE signal on neurites that were still intact (Fig. 5b). We chose the highest non-cytotoxic concentration (determined in the NeuriTox test) to test the core subset of inhibitors. In addition to the fluorescent endpoint, we also used Western blotting to quantify the amount of glycosylated proteins (= sialoproteins) (Suppl. Fig. 4c). Both endpoints were affected in a similar way by the test compounds, but the effects on the Western endpoint were less pronounced than in the fluorescent imaging assay (Fig. 4c, Suppl. Fig. 8b). According to this assay (termed here NeuroGlycoTest), cII inhibitors did not cause significant disturbances of proteostasis; some cI and cIII inhibitors showed clear effects. At the test concentration used, none of the inhibitors reduced cell viability, and only antimycin reduced the neurite area by more than 25% (Suppl. Fig. 4a). This suggests that effects in the NeuroGlycoTest were not only a passive consequence of neurite loss. In summary, these data suggest that changes in proteostasis are consistent with changes in the KE4/AO. However, the assay chosen here has a low throughput, and will require further work and optimization to be incorporated in a testing battery. It will also need to be compared to other potential proteostasis assays. At present, there is no consensus how to quantify this KE, and exploratory approaches as the one presented here are necessary. We contributed to this exploration by choosing two alternative assays within this study.

To assess a second endpoint of proteostasis, proteasomal function (enzymatic activity of the proteasome) was investigated in the same exposure scenario as for the NeuriTox test. To achieve this, we exposed cells to a cell-permeable substrate that becomes fluorescent upon cleavage by the proteasome. Quantification of the fluorescence can then be taken as measure of cellular proteasome activity (Fig. 5d). We observed indeed a concentration-dependent decrease in proteasomal function for all examined substances. However, this occurred only at concentrations that affected also the overall cell viability. The only exception was rotenone, which reduced proteasome function at non-cytotoxic concentrations (Fig. 5e). We conclude on this assay that it lacks sensitivity to measure effects on proteostasis that occur in still viable cells, and this approach was therefore not further pursued.

### Investigation of transcriptomic changes as biomarker for intermediate key events

**Fig. 6:**
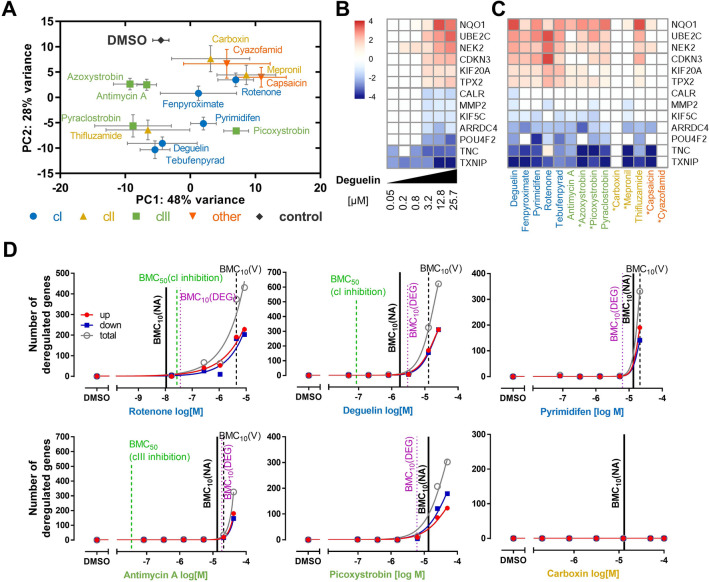
Investigation of altered mRNA transcripts as biomarker for mitochondrial respiratory chain impairment. **a** LUHMES cells were differentiated for 48 h and subsequently treated for 24 h. Analysis of the transcriptome (mRNA expression) was performed using Biospyder’s TempO-Seq technique. Test concentrations were the highest non-cytotoxic levels according to the NeuriTox assay. For non-cytotoxic compounds within the testing range, the highest possible concentration, i.e., 100 or 50 µM, was chosen (marked *). To visualize the overall data structure, a principal component (PC) analysis was performed. The 100 most variable genes were used to calculate the coordinates of PC1 and PC2, replicate values were plotted as mean ± SEM from 3 independent differentiations. Color-coding was applied based on the compounds’ MoA (respiratory chain complex inhibition), the PCA axes dimensioning was adjusted according to the variance explained by the individual PCs. **b** Genes regulated by deguelin as typical complex I inhibitor with strong transcriptional response were identified for EC_10_V (13 µM) and for a 4 × lower concentration (3.25 µM). The 13 overlap genes were considered to be regulated by definitely non-cytotoxic concentrations. They were used for expression analysis over the entire concentration range (26–0.05 µM). Upregulation by deguelin was color-coded red, down-regulation in blue, using a diverging scale of log2 fold change values. Regulations that had an FDR-corrected p value > 0.1 were colored white; data were from three independent differentiations; DEG were determined vs DMSO controls. **c** The regulation of the “deguelin-sensitive genes” identified in **b**) was investigated for all 14 compounds (tested at their EC_10_V concentration, if this was not reached, the highest tested concentration was used. **b** + **c** The mean fold change in expression relative to control from three independent differentiations was color-coded: blue indicates down-regulation, red indicates up-regulation, and white indicates that the regulation had an FDR-adjusted *p v*alue > 0.1. Compound colors indicate their target (cI, cIII, cII, other). **d** Concentration–response analysis of significantly differentially expressed genes (DEG). As reference values, the BMC_50_ concentrations of the respective respiratory chain complex inhibition (assessed in LUHMES cells) as well as the EC_10_ of viability [BMC_10_(V)] and neurite outgrowth impairment [BMC_10_(NA)] have been added (vertical dashed lines). DEG were defined by > 1.5-fold regulation (0.59 on log2 scale) and an FDR-corrected p value < 0.05 (data from three independent differentiations). The dotted pink line indicates the “BMC_10_” of gene regulation [BMC_10_(DEG)]. To determine this value, the degree of gene expression homeostasis (DGH) was defined as DGH = 100-number of deregulated genes; with a lower limit of DGH set to 0. The concentration-dependent DGH values were used as input for an algorithm to determine benchmark concentrations (Krebs et al. 2020) [http://invitrotox.uni-konstanz.de/BMC/] and BMC_10_ values were retrieved (color figure online)

An entirely different approach to define altered proteostasis makes use of high content Omics technologies (transcriptomics or proteomics) that would identify multiple changes in an unbiased way. We used here changes of the transcriptome, as biomarker of cellular changes related (indirectly) to changes of protein functions and expression levels. The TempOSeq high-throughput technology was applied here to allow multiple sampling at multiple concentrations. Samples were obtained from cells exposed for 24 h to five concentrations of 14 MRC inhibitors. All test compound concentrations were anchored to the respective EC_10_V values from the NeuriTox assay, so that for each compound, one data set was obtained for the highest non-cytotoxic concentration. A principle component analysis showed that all compounds exhibited a response different from the negative control (0.1% DMSO) at their respective EC_10_V. There was no obvious clustering of compounds according to the mode of action (e.g., cIII inhibitors were widely spread) (Fig. 6a).

To reduce the complexity of the analysis, and to allow still some information on concentration–response behavior and on similarity of responses between compounds, we took the following approach: (i) a gene set was identified that qualified as typical of neuronal cI inhibition; (ii) we checked for a selected compound (deguelin) the concentration dependency of gene regulation for this set; (iii) we checked for all compounds at a given concentration (EC_10_V) the congruence of dysregulation (Fig. 6b, c).

To select a typical cI gene set, we chose deguelin as gold standard. This compound showed strong responses in all KE assays, is a well-characterized, typical cI inhibitor (Delp et al. 2019; van der Stel et al. 2020) and behaves less extreme than rotenone. We selected all 13 genes that were differentially regulated by deguelin at its EC_10_V (13 µM), and at the next lower concentration (3.25 µM), to make sure that no unspecific indicators of cell death were included. This gene set included NQO1 (typical oxidative stress response) as usual suspect. It also comprised several genes related to microtubules (motor proteins and centrosome/spindle-related proteins), which suggests that a general disturbance of proteostasis occurred (Supp Fig. 5). Concentration–response analysis showed that 11 out of 13 of these genes were not significantly regulated up to 0.8 µM deguelin, but clearly upregulated at 3.25 µM. Above this concentration, the extent of regulation more or less reached a plateau, suggesting that these genes are not related to cell death (which was triggered in our system at the highest test concentration of 25 µM (Fig. 6d).

In a second step, we checked whether these genes were also affected by the other MRC inhibitors. All compounds active in the NeuriTox assay (all compounds, but capsaicin, mepronil, and cyazofamid) showed a dysregulation pattern that was very similar to deguelin. These data indicate that mitochondrial inhibitors with different modes of action show some similarities in their proteostasis response (Fig. 6b, c). Recently, indeed, a link has been found that links mitochondrial stress to the cytosolic stress response (Fessler et al. 2020). This pathway involves activation of the transcription factor CHOP and, therefore, may explain the gene regulation response triggered by mitochondrial inhibitors. Another way to relay such signals may involve reactive oxygen species produced by mitochondria and acting on cytosolic stress response systems (Krug et al. 2014).

Many more analyses would be possible, and we make all data available for this purpose (see suppl. Excel file). Our main intention here was to check the overall data structure and then to see whether a simple measure of the transcriptome response may be used as KE assay. We chose the overall number of differentially regulated genes (DEG) as potential endpoint, and used these data for curve fitting and determination of a benchmark concentration (BMC) of gene regulation [i.e., a concentration that may be regarded as threshold for gene regulation/disturbed proteostasis, BMC_10_(DEG)]. This concentration was designated here BMC_10_(DEG), and it was compared to the BMC_10_ for viability, for neurite growth, and for MRC complex inhibition (Fig. 6d, Suppl. Fig. 6).

The general cell viability was always the least sensitive endpoint. The question of whether neurite data or transcriptomics data were more sensitive to indicate potential toxicity was particularly interesting. For deguelin, both endpoints were in the same range, but cI inhibition occurred at considerably lower concentrations. This means that the specific KE4/AO endpoint (neurite damage) correlated with transcriptome changes and disturbed proteostasis; and that a certain extent of MRC inhibition neither triggered gene regulation, nor neurotoxicity.

For rotenone, neurite damage was the most sensitive endpoint. It occurred at < 50% cI inhibition, and before genes were deregulated. This data set supports an outlier role for rotenone, possibly due to a second target, different from cI. The situation for antimycin A was similar to the one for deguelin. When looking at all other compounds, some heterogeneity was apparent, as for some the gene regulation and for others, the neurite response was the more sensitive endpoint. In general, these measures were very often within a less than threefold variation (Fig. 6d, Suppl. Fig. 6).

From these data, we conclude that high-throughput transcriptomics, used for the determination of the number of DEG, may be used as endpoint to assess potential neurotoxicity, and that it is a biomarker indicating disturbed proteostasis. The data clearly indicate that no-effect levels exist for gene regulation and that this endpoint is not necessarily over-sensitive. Data from more systems will be required to decide on the overall sensitivity and specificity of this endpoint, and readout optimizations may be obtained by selections of particular gene sets like stress response genes (Wink et al. 2018; Zgheib et al. 2018), differentiation-related genes (Dreser et al. 2020), or good collections of genes related to the specific KE or AO under investigation.

### Investigation of the mitochondrial membrane potential (MMP) and resazurin reduction impairment to assess KE2

**Fig. 7:**
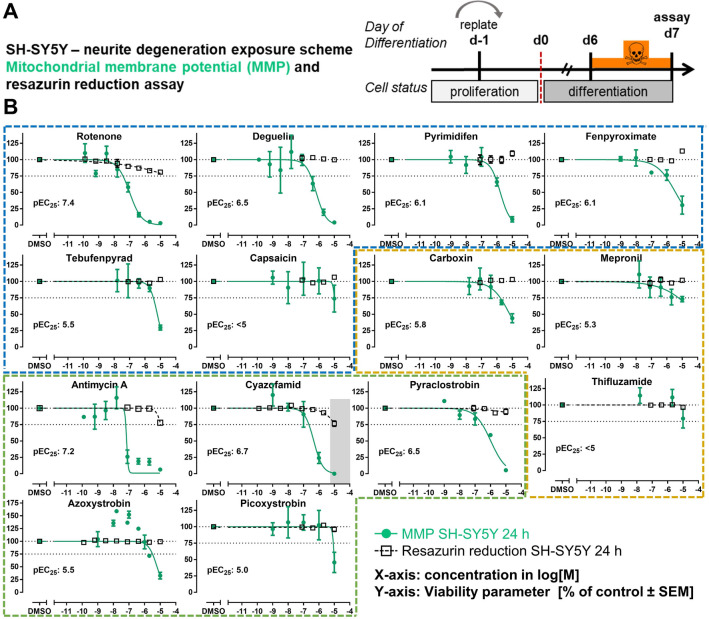
Investigation of mitochondrial membrane potential (MMP) and resazurin reduction impairment to assess KE2. **a** SH-SY5Y cells were differentiated for 6 days and subsequently treated for 24 h. Endpoints were determined using either rhodamine-123 for MMP (green) or resazurin reduction for overall viability (black). **b** Concentration–response graphs for the subset of cI (blue), cII (yellow), and cIII (green) inhibitors, ordered according to the compounds’ potency within their MoA group. Data are means ± SEM from three independent experiments. The numbers in the graphs indicate the EC_25_ for MMP (crossing point of MMP curve fit with dotted line at 75%). These data are used for downstream data comparisons as KE2 output. pEC_25_: − log[M] concentration where MMP was reduced by 25% (color figure online)

According to the AOP:3, the KE4/AO can not only be triggered by KE3 (disturbed proteostasis), but also (directly) by KE2 (mitochondrial dysfunction). In our approach of working backwards along the AOP, we used assays relevant to KE2. Measurements of the mitochondrial membrane potential (MMP) were used here as main approach, as the MMP is a well-established proxy of mitochondrial function. For this assay, we used SH-SY5Y cells, as the endpoint is well established for this test system (Feng et al. 2020; Gustafsson et al. 2004; Kim et al. 2017; Pakrashi et al. 2020). The cells were cultured and exposed for 24 h as for the neurite degeneration assay (see Fig. 3). General cell viability was assessed in parallel by the resazurin reduction test (Fig. 7a), and none of the MRC inhibitors was bluntly cytotoxic (resazurin reduction was always > 75% of control values). In this setting, the MMP was affected drastically by many compounds at non-cytotoxic concentrations. All confirmed cI and cIII inhibitors (excluding capsaicin) decreased the MMP by at least 60%. The cII inhibitors showed less pronounced effects (none reached a 60% inhibition, but carboxin reached at least 50%). Thus, this assay proved to be a very sensitive endpoint (Fig. 7b).

To complement these data, we also used LUHMES cells to measure resazurin reduction (Suppl. Fig. 7a). This endpoint measure was not reduced by MRC inhibitors (at concentrations below those also affecting the neurites). In fact, the resazurin signal often was rather increased. This is a frequently observed artifact, most likely linked to changes of cellular metabolism, like the increase of the reducing agent NADH (Blacker and Duchen 2016) (Suppl. Fig.7b). As the MMP assay is not established for LUHMES cells, we explored whether intracellular ATP levels could be a sensitive indicator of mitochondrial dysfunction. However, a decrease was not observed at concentrations lower than those affecting neurite morphology (Suppl. Fig. 7b; synoptic overview in Fig. 4). In summary, the SH-SY5Y MMP assay proved to be most suitable to test mitochondrial dysfunction. We observed earlier that different cell types can give different MMP data and each test system requires a lot of method adaptations to yield robust results (van der Stel et al. 2020). The LUHMES assay did not work under conditions that were optimal for SH-SY5Y cells. An alternative mitochondrial function assay in LUHMES cells relies on the comparison of test results in glucose vs galactose medium. We found for some compounds (rotenone, deguelin, and antimycin) that ATP measurements in galactose medium may be an indicator of mitochondrial function (Delp et al. 2019). This assay was not used here, as the SH-SY5Y system already provided a suitable KE2 assay, and as the interpretation of the test battery becomes extremely complex, when a new test system/medium composition is used for one KE, but not for others. Given the scope and complexity of the study with so many inhibitors, concentrations, and replicates, the additional effort to run all assays in two media was not possible within the project.

### Quantification of respiration in intact and permeabilized neurons to assess initial KEs.

**Fig. 8:**
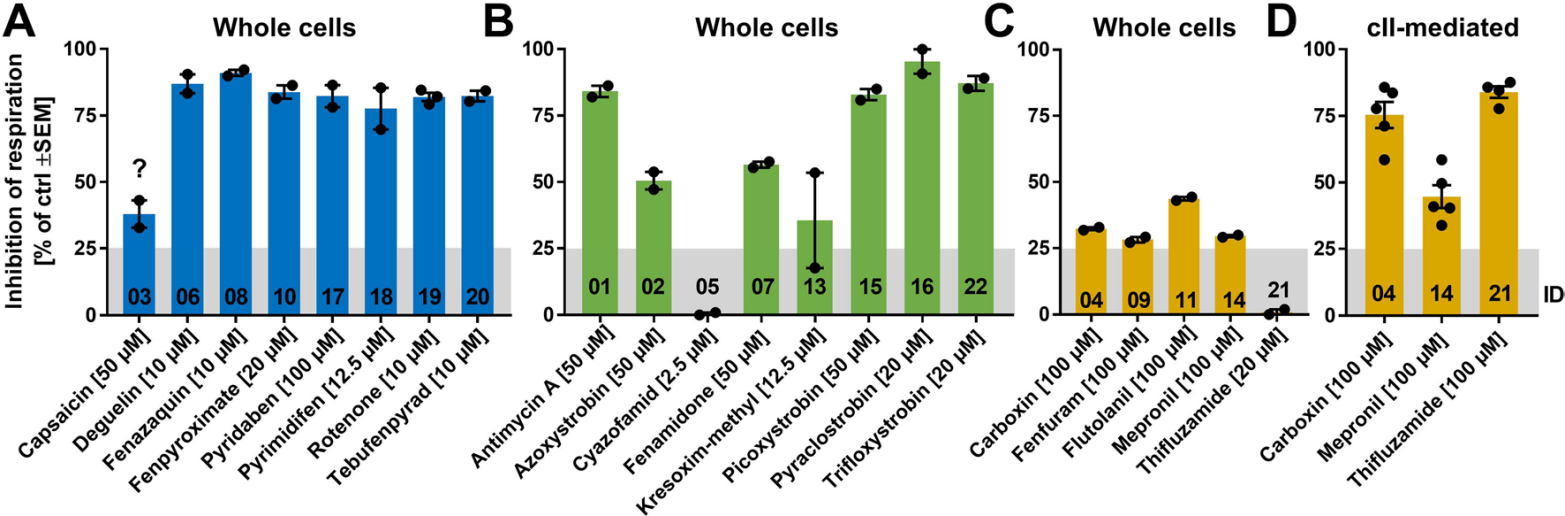
Quantification of respiration of intact and permeabilized LUHMES cells. **a–c** As assay to assess MIE, KE1, and KE2, intact (whole cells) d3-differentiated LUHMES cells were acutely treated with the indicated inhibitors at their highest non-cytotoxic concentration and changes in mitochondrial oxygen consumption rate were quantified. Compounds are grouped and color-coded according to their MoA (inhibitors of cI, III, and II in blue, green, yellow). **d** Inhibition of cII activity by cII inhibitors was assessed using permeabilized LUHMES cells as depicted in Suppl. Figure 7. The assay specifically assesses cII activity. The numbers at the bottom of the bars indicate the compound ID. Bars represent means ± SEM, and each point represents the result of an independent experiment. The gray area is the negative control noise band of the assay as defined in ***(***Delp et al. 2019***)***, i.e., inhibition within this range is regarded to be insignificant (color figure online)

In the next step, we moved to assays that can inform on initial KE of the AOP. We chose to measure the activity of the MRC using the oxygen consumption as endpoint. This assay can be considered as providing (indirect) information on the MIE/KE1 (binding to MRC complexes and inhibition of their activity). LUHMES cells were tested at the highest non-cytotoxic concentration (determined from the NeuriTox assay, Fig. 2). Investigation of the total mitochondrial respiration of intact cells showed that all cI inhibitors (except capsaicin) had strong (> 75%) inhibitory effects (Fig. 8a). Capsaicin showed a weak response. For further confirmation, we investigated the biochemical activity of the compounds more directly in permeabilized cells. In this assay, electrons can be fed specifically into cI of the respiratory chain (Suppl. Figure 8a), and all cI inhibitors were confirmed to block cI to a high degree, with the only exception of capsaicin (no significant response at 50 µM) (Suppl. Fig. 8b, d). These data are fully concordant with our data from liver and kidney cells, where capsaicin showed only faint mitochondrial effects, and had an IC50 of > 200 µM for inhibition of cI (Fig. 8a) (van der Stel et al. 2020).

For the investigated cIII inhibitors, 4 of 8 had strong inhibitory effects (antimycin and the strobins), and 3/8 showed intermediate inhibition. Cyazofamid was inactive (Fig. 8b). The cIII inhibitors that had strong effects on respiration of intact LUHMES neurons also decreased specific cIII activity to a similar extent in permeabilized cells (and none of them affected cIV activity; data not shown). Cyazofamid was not active at all in the more direct cIII assay, using a cIII electron donor (Suppl. Fig. 8d). This observation in neurons agrees well with our earlier findings in HepG2 cells, where this compound was identified as being mis-classified and indeed being an uncoupler (van der Stel et al. 2020). The uncoupling action was also found here for neuronal mitochondria at a cyazofamid concentration of 50 µM (Suppl. Fig. 8d).

The cII inhibitors all showed a weak inhibition of mitochondrial respiration, even at concentrations of 100 µM (Fig. 8c). As it was important for the interpretation of our study to clarify whether these compounds inhibit at all cII of neuronal mitochondria, we investigated the biochemical activity of some cII compounds in permeabilized cells. In this assay, electrons were fed directly into cII of the respiratory chain (Suppl. Fig. 8a). Carboxin and thifluzamide showed indeed a strong inhibition of cII (Fig. 8d), without affecting cI, III, or IV activity (data not shown). Mepronil (at 100 µM) was a significant, but weak inhibitor in this assay (Fig. 8d), but it also weakly affected cI (Suppl. Fig. 8c). This is fully in agreement with data from HepG2 cells, where 50% inhibition was only observed at concentrations > 100 µM, and a low specificity was noted (van der Stel et al. 2020). It is important to note that at least some of the purported cII inhibitors did affect their cognate target, but their impact on overall respiration was attenuated in intact cells, probably by compensation with electrons from cI. This would explain the low or absent neurotoxicity of these compounds.

One reason for different effects of inhibitors may be the complexity of the MRC. Its complexes are huge multi-protein assemblies (cIV, III, and I consist of 13, 22, and 45 protein subunits, respectively), their function can be inhibited at many different sites (Guo et al. 2018). Inhibitors may interfere i) with the binding of the substrate (e.g., NADH) to the complex, ii) with the electron transfer within the complex, or iii) with the transfer of the electrons from the complex to the next substrate (e.g., cI to Q10) (Friedrich et al. 1994; Ino et al. 2003; Okun et al. 1999). The site at which rotenone interacts with cI is known, and cryo-electron microscopy structures are available (Fendel et al. 2008). Recently, it has been shown that deguelin, a rotenone analog, binds to the same site (Troger et al. 2020). For cIII, three inhibitor-binding sites have been identified (Qi, Qo, and NQ site) (Hagras and Stuchebrukhov 2016; Lai et al. 2005), but it is not clear whether more binding sites exist.

### Qualitative overview of KE coherence

We used the data sets from our AOP-guided testing scheme, to compare the concordance across different assays. Our approach was to analyze how far initial KE activation agreed with activation of KE4/AO (assayed in the NeuriTox assay). For this comparison, we considered a fixed concentration of 50 µM, as might be used in a screening approach. KE4 was considered to be triggered (“ + ”), when a compound showed ≥ 25% effect; otherwise, it was regarded as inactive (“O”). Having set this anchoring point for our comparisons, we checked then whether various other KE assays (for KE1-3) were concordant with KE4 or not. All cI and cIII inhibitors caused neurite toxicity at 50 µM (sometimes with accompanying cytotoxicity), and we considered all of them as hits in the KE4 assay. Of the cII inhibitors, thifluzamide was also a hit, while other compounds (carboxin, mepronil, and cyazofamid) were non-hits. All cI inhibitors showed a coherent KE3 assay activity (proteostasis assay) and KE2 assay activity (MMP assay). As test for MRC inhibition, we used LUHMES-based respirometric data (in intact and in permeabilized cells) (Fig. 8, Suppl. Figs. 8 and 10a), and also there, concordant activity (with KE4) was observed. Thus, in this fixed (50 µM) concentration (virtual) screen, a high concordance was noted for cI inhibitors. A more or less similar conclusion could be derived for the cIII inhibitors and for the active cII compound thifluzamide (with some inconsistencies on the KE3 assay). Conclusions on the non-hits were difficult to derive, as effects for these low-potency compounds were often at the borderline of the detection range and of the solubility in the assays. Altogether, this initial comparison draws a consistent picture (at least for positive compounds): it confirms what has been described in the original AOP:3 for rotenone and MPP + , now for more cI inhibitors and extending it to cIII inhibitors (Suppl. Figure 9). In summary, this initial overview suggested that there is a consistent and concordant activation of the AOP throughout all KE up to the AO. However, this initial overview did not consider potential potency differences and it also did not account for unspecific effects due to cytotoxicity.

Data in support of many AOP have been assembled in such a way, as they had to be derived from many publications and assays, from approaches using different concentration ranges and from publications not always providing full concentration–response data sets. We tested next whether the picture may change, when more comprehensive data are available for a more quantitative comparison.

### Synoptic overview of the sensitivities of different assays along the AOP

Concentration–response data from various assays specifically assembled to probe AOP:3 KEs, allowed us to compare KE activation (using the respective EC_25_ concentrations) quantitatively. We used again the neurite toxicity assay (probing KE4/AO) as anchoring point, and considered the ratios of data on KE4/KE3, KE4/KE2, and KE4/KE1 for the subset of compounds tested in all assays.

**Fig. 9:**
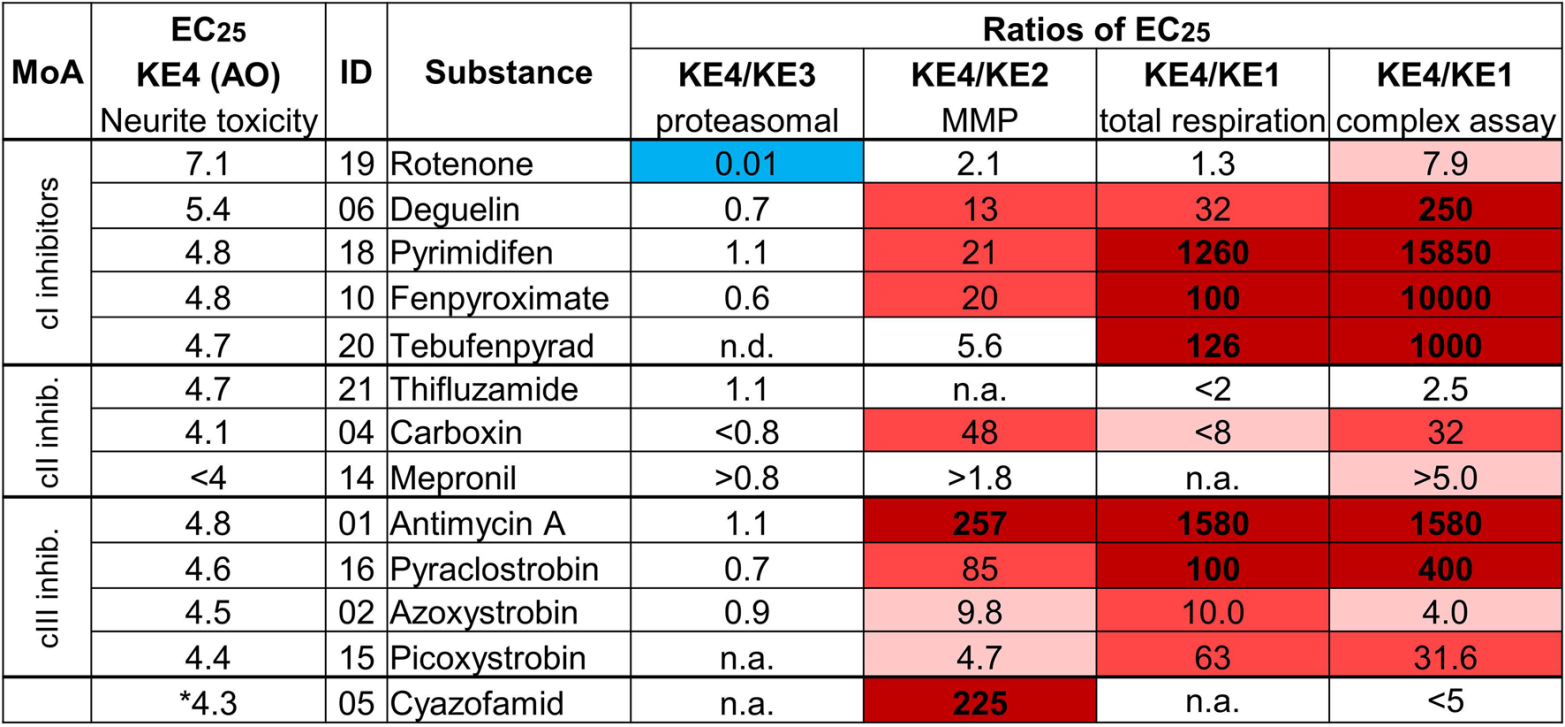
Synoptic overview of the sensitivities of different assays along the AOP. Synoptic overview of the EC_25_ concentrations (in − log[M]) of different assays along the AOP for the subset of 14 toxicants which have been characterized in depth. Anchoring point is the EC25 concentration for neurite outgrowth impairment of the NeuriTox test for KE4 (in vitro proxy for the AO). EC_25_ concentrations of other KE assays were displayed as ratio (e.g., KE4/KE1) and colored in faint red if > 3, in red if > 10 and dark red if > 100 (i.e., when upstream KE1-3 assay was more sensitive than KE4) and blue if < 0.33 (i.e., upstream KE3 was less sensitive than KE4) or white for ratios between 3 and 1/3. Note: data on respirometric inhibition were retrieved from HepG2-based assays (van der Stel et al. 2020); n.a.: ratio could not be determined due to low effects; n.d.: not determined. Cyazofamid was in this study not confirmed to be a cIII inhibitor

The KE4/KE3 ratio was mostly in the range of one. From this, one may conclude that the potency of compounds in the KE3 assay is largely similar with that in the neurotoxicity assay, and that, therefore, KE3 assays could be quantitatively predictive for the AO. However, there was one exception and one general caveat. Rotenone was the outlier compound in this comparison, as it affected neurotoxicity (KE4/AO) 100 times more potently than proteostasis (KE3). This effect was not observed for deguelin, which otherwise has many similarities to rotenone. The general caveat is that many of the compounds triggered relatively unspecific cytotoxicity (in the two assays). Since both tests (for KE4 and KE3) are cell-based (LUHMES), the cytotoxicity data are similar, and they result, therefore, in a ratio of 1. Some of this result is, therefore, not suitable for far-reaching conceptual interpretations (Fig. 9). It also needs to be noted that AOP:3 allows for KE3 to be bypassed, and in the models chosen, there was possibly a direct activation of KE4 by KE2.

The KE4/KE2 ratio was next in the synoptic comparison: cI and cIII inhibitors showed overall quite similar patterns: the mitochondrial membrane potential was mostly impaired at 1–2 orders of magnitude lower concentrations (10–260 times lower) than those found for KE4. This is an important observation for the strategy of using KE assays instead of AO assays. In the context of quantitative hazard assessment, and an in vitro-to-in vivo extrapolation (IVIVE) the predicted equivalent oral doses would differ by 1–2 orders of magnitude, when KE4 or KE2 assay data are used as point-of-departure (POD) (Fig. 9). The difference is unlikely to be explained by differences in free fractions of the test compound in the media of the different assays, as both the LUHMES system and the SH-SY5Y system had very low protein and lipid concentrations (no serum) in the medium (Krebs et al. 2020).

We also examined how the MMP (KE2) assay related to the data on MRC inhibition. As inhibition of cell respiration/MRC complex activity was measured in neurons only for fixed concentrations, we used our previously published data from non-neuronal cells (van der Stel et al. 2020). This assumption of similar biochemical effects on the MRC in various cell types appeared justified, based on the strong concordance between respirometric data derived from HepG2 cells and RPTEC/TERT1 cells (van der Stel et al. 2020), as well as a good correlation of the concentration–response data for rotenone and deguelin in LUHMES and HepG2 cells (Suppl. Fig. 10a), and the good agreement of the single concentration data on many compounds in neurons, HepG2 and RPTEC cells (van der Stel et al. 2020). The MMP assay was clearly less sensitive for cI inhibitors than the more upstream MRC complex assay. There was some variation for cIII inhibitors, but on average, the sensitivity of this group of test compounds was similar, and the same applied to cII inhibitors (Suppl. Fig. 10b). Thus, the offset of KE assays might depend on the type of compound tested, or on the exact target. Notably, the AOP:3 is strictly defined only for cI inhibitors (Terron et al. 2018). For such compounds, sensitivities decreased from KE1 to KE4 by about a log step per KE.

To further check this, it was interesting to move further along the AOP: the KE4 was compared to the sensitivity of the cellular respiration assay (a proxy for MRC complex inhibition; KE1). It was, for some substances, similar to the KE2 assay, and for some others, it was another ten times more sensitive than the MMP assay. When permeabilized cells were used, a further increase in sensitivity was observed for most cI inhibitors, while cIII inhibitors gave mostly similar results for the two KE1 assays (using intact or permeabilized cells) (Suppl. Fig. 10c; Fig. 9).

In summary, these comparisons of KE assays showed that the general trend was definitely that the concentrations triggering earlier KE were much lower (2–4 orders magnitude) than those triggering later events. This suggests that the AOP has a strong element of counter-regulation and buffering (Leist et al. 2017), so that triggering of an early KE does not always lead to the AO (Fig. 9).

This overall picture was fully confirmed, when we looked at further compounds, for which not all data (e.g., on KE3) are available. Also for the cI inhibitors fenazaquin and pyridaben, the ratio of the NeuriTox assay and the cell respiration assay was 40–60, and the ratio of NeuriTox to the cI assay in permeabilized cells was ≥ 800. For the additional cIII inhibitors (fenamidone, kresoxim-methyl, and trifloxistrobin), the KE4/KE1 ratio was ≥ 10 (when cell respiration was considered, and remained in a similar range, when permeabilized cells were used (Suppl. Fig. 11)). Thus, for cI inhibitors, permeabilized cells yielded much lower EC_25_ values than those obtained in live cells, while for cIII inhibitors, there was no large difference between the data from these two assays. For cII inhibitors, most responses were weak and in a similar concentration range. The only exception was carboxin (KE4/KE2 ratio: 48 and KE4/KE1 ratio: 32), which behaved like some cIII inhibitors (Fig. 9; Suppl. Fig. 11).

The ranking of the KE sensitivities was not surprising, as it is quite consistent with the AOP concept that initial KE may be activated by lower toxicant concentrations, and that more and more disturbance is required to trigger the final adversity. However, a few studies are available on this phenomenon, and such data are urgently required to improve strategies for quantitative hazard prediction and risk assessment based on integrated approaches to testing and assessment (IATA). Two very important issues are, e.g., in how far early KE assays, or even data from assays related to MIEs can be used for IVIVE. For instance, the ToxCast program has been initially focused on MIE-related assays, and the correlation of these with AO has proven to be low (Thomas et al. 2012). However, in some biological areas, e.g., endocrine signaling, also very good results have been obtained (Browne et al. 2015; Kleinstreuer et al. 2018). An explanation for this may be that some AOP, and certain sets of chemicals show large sensitivity changes for KE activation, while others may yield similar, highly consistent PoD for all early and late KE. Also in our study, we see that even within one AOP, the ratio of KE4/KE1 is larger for cI inhibitors than for cIII inhibitors.

Concerning the different assay sensitivities, it was particularly striking that there appeared to be a clear offset between MRC inhibition tests in intact cells and in permeabilized cells. As all test compounds are quite hydrophobic, the cell permeability is unlikely to account for this difference. For a clear overview of the situation, we compared the data directly to one another (Suppl. Fig. 10d). This overview, generated on the basis of our previously published data (van der Stel et al. 2020), reveals two interesting features: (a) the group of cI inhibitors is rather more potent than the group of cIII inhibitors (with the notable exception of antimycin A); (b) for cIII inhibitors, the whole-cell assay and the cIII assay in permeabilized cells yielded very similar data, while all cI inhibitors were more potent on the cI assay than in the whole-cell respiration assay. The cII inhibitors were 2–3 orders of magnitude less potent on their complex, than the cI inhibitors on their cognate target. Such potency differences may play a role. However, also a biological rationale may explain the different behavior: cI and cII inhibitors may be less potent in cellular assays, as both complexes are redundant and may be bypassed by the respective other one. This is not the case for cIII, which is absolutely necessary and unreplaceable for respiration to take place. In the permeabilized cell assays, the cI and cII were fed electrons from complex-specific donors, and could therefore not be bypassed. Thus, a higher sensitivity might have been observed in this case. Another potential explanation is that the permeabilized cell assay quite clearly addresses KE1 (inhibition of the MRC). The whole-cell assay addresses to some extent general mitochondrial dysfunction, and may therefore be considered as KE2 or as an assay at least partially addressing KE2. As this positions the whole-cell assay somewhat more downstream in the AOP, it may also explain a sensitivity difference (see positioning in Fig. 1).

## Conclusions and outlook

In this study, we set out to explore, (i) whether the AOP:3 (Terron et al. 2018) may be expanded by inclusion of inhibitors of mitochondrial cII and cIII, (ii) whether it could be applied as basis for hazard assessment of industrial and environmental chemicals, such as the set of pesticides chosen here, and (iii) whether rules could be defined on how to use data from KE assays or transcriptome analysis to predict in vivo neurotoxicity. The data generated here lead to a number of important conclusions:

First, the test compounds showed a very heterogeneous behavior, although they were all selected for being classified in the literature as mitochondrial respiratory chain inhibitors. For instance, our MRC complex inhibition data showed that mepronil and capsaicin were mis-classified. These compounds are therefore not suitable as positive controls for MRC inhibitors in subsequent studies. However, also *bona fide* inhibitors showed some unexpected behavior: the cII inhibitors of our study had low potency, and they were difficult to compare to the other compounds. These compounds have been developed to target succinate dehydrogenase of fungi (Lewis et al. 2016), and possibly, there are considerable species differences between the fungal and human protein core subunits. More thorough biochemical studies will be required here. There were many similarities between the behavior of cI and cIII inhibitors, so that an expansion of AOP:3 to cIII inhibition could be justified. However, the situation is complicated by the fact that there was large heterogeneity within the cI and cIII inhibitor groups. For instance, rotenone differed from tebufenpyrad, and antimycin A differed from the strobilurins in their response patterns. It was striking that only some compounds showed a specific neurotoxicity (e.g., rotenone), i.e., a damaging effect on neurites without overall loss of neuronal viability. The mechanistic reasons are at present not well understood, but it is likely that additional targets of the compounds are involved. The potent neurotoxicity of rotenone may be due to additional effects of this compound, e.g., on glycolysis. However, decades of use of this tool compound have not resulted in any data supporting this hypothesis. An alternative explanation could be that rotenone affects other processes that act synergistically with mitochondrial inhibition. There are, for instance, several reports on rotenone’s effects on microtubules, affecting, e.g., mitochondrial transport or kinetochore assembly (Brinkley et al. 1974; Cabeza-Arvelaiz and Schiestl 2012; Passmore et al. 2017; Ren et al. 2005; Srivastava and Panda 2007). The largely different potencies and specificities for functional effects within cI inhibitors suggest that modulatory events play an important role in AOP:3, and that each compound may affect them in different ways (allowing for more or less counter-regulation or synergy of events). One potential strategy to follow up on this would be to establish a quantitative AOP, based on system biology principles (feedback loops and modulatory events) and to feed it with more time-dependent data sets. For such work, the compound set used here could be of high interest, as its heterogeneous behavior should be explained by such a model. This is well in line with the intended use of AOP (or AOP networks) for risk assessment: they should not only work for few, “mechanistically-clean” tool compounds, but for real-life chemicals, with a more “dirty” target profile. Within a more comprehensive risk assessment exercise, also toxicokinetic and biokinetic behavior would be taken into account, and then, the results of the KE assays should be related to the in vivo toxicity data. An example of this will be given in an extensive read-across study on the rotenone-deguelin couple to be published within the OECD IATA case study program by the end of 2020.

Second, we found here evidence that the key events of the AOP (or possibly also of other AOP) are so broadly defined that they do not give sufficiently defined guidance on which is the most suitable KE assay to use for a test battery. The KEs, in their presently wide definition (like “mitochondrial dysfunction” or “disturbed proteostasis”), are very useful for descriptive AOPs with the purpose to sort literature data and to give a mechanistic rationale why a certain mechanistic endpoint is linked to an adverse outcome (Leist et al. 2017). For such applications, the large “bins” (combining several biochemical mechanisms in one KE) allow conceptual categories to be formed, that are easy to describe, rationalize, and remember, and that allow broadly applicable rules to be identified (e.g., any type of mitochondrial dysfunction is closely linked to a neurotoxicity hazard). For fully quantitative assessment, the large KE categories are difficult to handle. They may not account sufficiently for the individual and specific properties of each chemical. The exact type of disturbed proteostasis and the exact type of mitochondrial dysfunction may have a large impact on the adverse outcome and on the concentration triggering it. Moreover, the broadly defined KE hardly account for biological subtleties, such as differences of mitochondria from cell type to cell type (Borchard et al. 2018; Kappler et al. 2019). There is also a second issue with broadly defined KE: they allow for mechanistically different types of assays to assess them. Each of these assays may yield different hits, or give varying potencies for a given compound (as shown here e.g., for KE1 assays). It is an important and formidable task for the future to select the best-predictive assays to assess each KE and/or to understand how data from KE assays should be combined for general hazard assessment.

Third, our synoptic overview clearly showed that the KE assays produce largely (several orders of magnitude) different points-of-departure. The early KEs were more sensitive to test compounds than late KE/AO. This triggers the question how the KE assays are to be combined for a meaningful in vitro-to-in vivo extrapolations. This situation also implies that not just any KE can be measured if quantitative risk assessment is desired. One may argue that early KE assays are over-sensitive, as their triggering does not always lead to an AO. On the other hand, they could constitute an important alert that can then be followed up with more detailed investigation. In this context, further studies are required on modulating and counter-acting factors, as they may differ from cell to cell or from one human individual to another. Moreover, exposure time may need to be considered, as triggering of early KE may be tolerated by cells (and tissues in vivo) for short periods, but it may result in toxicities after prolonged or repeated exposure.

We addressed here the role of exposure time, by comparing short-term exposure and repeated/prolonged exposure over several days. In this context, it is important that prolonged exposures are experimentally difficult, sometimes even impossible in many test setups. As far as robust test conditions could be established, we did not find major differences between shorter and longer exposures, when neurotoxicity was used as endpoint. For exposures over several weeks, as may be relevant for human pesticide exposure, novel 3D organoids need to be used (Brüll et al. 2020), as conventional cultures have a limited “shelf-life”. Another important modulating condition is the metabolic situation. We have found earlier that substitution of glucose in the culture medium by galactose can drastically increase the sensitivity of cells to cI and cIII inhibitors (Delp et al. 2019; van der Stel et al. 2020). This relatively artificial culture environment (brain cells are not exposed to galactose) has been avoided in our study strategy to allow direct transfer of in vitro findings to the in vivo situation. However, we suggest to consider this approach for future studies to span the whole range of potential metabolic situations that may occur in neurons. Under galactose conditions, there would also be considerably less offset in the PoD for various KE.

In summary, the findings of our study suggest that testing along the KE of an AOP can give important guidance on what type of hazard is to be expected. Our data also suggest that the use of KE data for quantitative risk assessment is not straight-forward, and will most likely need to be much more thoroughly explored in many cases. For a qualitative judgment, testing along AOP KEs will be helpful, especially, if a prediction model can be developed that integrates the data from the assays into an overall prediction. Such a process requires many in vivo data to be available for calibration, and it is being explored at present for the skin sensitization assay (Gilmour et al. 2020).

A side aspect of our study is that we explore the use of gene expression data to support KE activation. Although we present here only an initial and superficial analysis, there is already an important finding: the NOEL (or BMC_10_) of gene expression falls within the range of other KE test data. Thus, this omics approach is by no means over-sensitive. This means that it may be possible to define sets of genes that indicate the activation of a KE within a relevant concentration range. One approach to do this is to apply weighted gene co-regulated network (WGCN) analysis (Copple et al. 2019). If this is calibrated in a way to find a threshold of activation that correlates with adversity, WGCN analysis might provide a general method to test for AOP activation. This points to important follow-up work: finding the right time point to assay the transcriptome change, defining the relevant WGCNs, and defining their threshold of adversity. The set of compounds studied here may be an important toolset for this approach.

## Supplementary Information

Below is the link to the electronic supplementary material.

Supplementary file1 (XLSX 16231 KB)

Supplementary file2 (PDF 20551 KB)

## Data Availability

TempO-Seq data have been deposited as described in methods and supplementary information. Other data are available upon reasonable request.

## Abbreviations

AO: Adverse outcome
AOP: Adverse outcome pathway
ATP: Adenosine triphosphate
cAMP: N6,2′-O-dibutyryladenosine 3′,5′-cyclic monophosphate
cI–V: MRC complex I–V
CNS: Central nervous system
Cyt c: Cytochrome c
ETC: Mitochondrial electron transport chain, i.e., MRC
FAD(H_2_): Flavin adenine dinucleotide (FAD: oxidized, FADH2: reduced)
FCCP: Carbonyl cyanide-4-(trifluoromethoxy)phenylhydrazone
GDNF: Glial-derived neurotrophic factor
KE: Key event
KER: Key event relationship
MIE: Molecular initiating event
MoA: Mode of action
MPP: 1-Methyl-4-phenylpyridinium
MRC: Mitochondrial respiratory chain
NA: Neurite area
NAD(H): Nicotinamide adenine dinucleotide (NAD: oxidized, NADH: reduced)
O_2_: Oxygen
OCR: Oxygen consumption rate
Q: Ubiquinone or coenzyme Q
ROS: Reactive oxygen species
TCA: Citric acid cycle or Krebs cycle or tricarboxylic acid cycle
V: Viability
