# Supplementary material for: Neurotoxicity and underlying cellular changes of 21 mitochondrial respiratory chain inhibitors
Source: Arch Toxicol. 2021 Jan 29;95(2):591–615. doi: 10.1007/s00204-020-02970-5 (PMC7870626; doi:10.1007/s00204-020-02970-5)
Supplement: Supplementary file 2 — Supplementary file2 (PDF 20551 KB) [file 204_2020_2970_MOESM2_ESM.pdf]

Neurotoxicity and underlying cellular changes of 21 mitochondrial respiratory chain inhibitors

Johannes Delp[1,2], Andrea Cediel-Ulloa[3,10], Ilinca Suciu[1,4], Petra Kranaster[1,4], Barbara MA Van Vugt-Lussenburg[5], Vesna Munic Kos[3,11], Wanda van der Stel[6], Giada Carta[7], Susanne Hougaard Bennekou[8], Paul Jennings[7], Bob van de Water[6], Anna Forsby[3,9], Marcel Leist[1]

1: Chair for *In Vitro* Toxicology and Biomedicine, inaugurated by the Doerenkamp-Zbinden Foundation, University of Konstanz, Konstanz, Germany

2: Cooperative Doctorate College InViTe, University of Konstanz, Konstanz, Germany

3: Swetox, Unit for Toxicological Sciences, Karolinska Institutet, Stockholm, Sweden

4: Konstanz Research School Chemical Biology (KoRS-CB), University of Konstanz, Konstanz, Germany

5: BioDetection Systems BV, Amsterdam, The Netherlands

6: Division of Drug Discovery and Safety, Leiden Academic Centre for Drug Research, Leiden University, Leiden, The Netherlands

7: Division of Molecular and Computational Toxicology, Amsterdam Institute for Molecules, Medicines and Systems, Vrije Universiteit Amsterdam, Amsterdam, Netherlands

8: National Food Institute, Technical University of Denmark (DTU), Lyngby, Denmark

9: Department of Biochemistry and Biophysics, Stockholm University, Stockholm, Sweden

10: Department of Organismal Biology, Uppsala University, Uppsala, Sweden

11: Department of Physiology and Pharmacology, Karolinska Institutet, Stockholm, Sweden

Table of contents

Suppl. Fig. 1: Overview of test compounds, their mode of action and literature background data	p. 2
Suppl. Fig. 2: Chemical structures of test compounds	p. 3
Suppl. Fig. 3: Comparison of neurotoxicity after acute and chronic exposure in two models	p. 5
Suppl. Fig. 4: Viability parameters and Western blots of the NeuroGlycoTest	p. 6
Suppl. Fig. 5: Background information on the genes found to be regulated in LUHMES cells by two non-toxic deguelin concentrations	p. 7
Suppl. Fig. 6: Concentration-response analysis of the number of differentially expressed genes	p. 8
Suppl. Fig. 7: Assessment of intracellular ATP levels and resazurin reduction in the NeuriTox test	p. 10
Suppl. Fig. 8: Schematic representation of the mitochondrial complex activity assay and data for cI and cIII inhibition	p. 12
Suppl. Fig. 9: Comparison of the sensitivities of different assays along the AOP	p. 13
Suppl. Fig.10: Overview of AOP-consistent triggering of key events	p. 14
Suppl. Fig. 11: Scatter plots comparing the sensitivity of different KE assays and their derivatives with each other	p. 15
Suppl. Data: Differential gene expression analysis results for the 14 compounds subset	p. 16 + separate file

ID#	Compound	Target	Cytotox. (1) U2OS	EC_{25} respiration impairment permeabilized (2)	intact (3)	Pesticidal use PubChem, IRAC, FRAC
03	Capsaicin (*)	cI	4.2	3.9	<5	Reppellent, kills mites and ants
06	Deguelin (*)	cI	<4.3	7.8	6.9	Not used. Piscicide
08	Fenazaquin	cI	<4	7.9	6.6	Fungicide, insecticide/ acaricide
10	Fenpyroximate (*)	cI	<4.3	8.8	6.8	Insecticide/ acaricide
17	Pyridaben	cI	<4	8.7	6.0	Insecticide/ acaricide
18	Pyrimidifen (*)	cI	4.0	9.0	7.9	Insecticide/ acaricide
19	Rotenone (*)	cI	4.5	8.0	7.2	Not used. Piscicide
20	Tebufenpyrad (*)	cI	<4	7.7	6.8	Insecticide/ acaricide
04	Carboxin (*)	cII	<4	5.6	<5	Fungicide
09	Fenfuram	cII	<4	3.9	<5	Fungicide
11	Flutolanil	cII	<4	4.9	<5	Fungicide
14	Mepronil (*)	cII	<4	4.7	<5	Fungicide
21	Thifluzamide (*)	cII	4.7	5.1	<5	Fungicide
01	Antimycin A (*)	cIII	4.0	8.0	8.0	Fungicide, insecticide/ acaricide, piscicide
02	Azoxystrobin (*)	cIII	<4	5.1	5.5	Fungicide
05	Cyazofamid (*)	cIII	4.0	<5	<5	Fungicide
07	Fenamidone	cIII	<4	5.7	5.9	Fungicide
13	Kresoxim-methyl	cIII	<4	5.9	5.4	Fungicide
15	Picoxystrobin (*)	cIII	<4	5.9	6.2	Fungicide
16	Pyraclostrobin (*)	cIII	<4	7.2	6.6	Fungicide
22	Trifloxistrobin	cIII	4.3	5.9	5.7	Fungicide

Suppl. Fig. 1: Overview of test compounds, their mode of action and literature background data.

In this study, a set of 21 literature-described mitochondrial respiratory chain complex I, II and III inhibitors (target), was used. A subset of 14 compounds (*) was tested in a comprehensive test battery and the remaining eight compounds were tested in fewer assays. (1) General/ unspecific cytotoxicity was assessed in osteosarcoma cell (U2OS) cultures. (2) Impairment of respiratory chain complexes was assessed in permeabilized HepG2 cells (van der Stel et al. 2020) with specific measurements of cI activity for cI inhibitors, and cII/III activity for cII and cIII inhibitors. (3) Inhibition of respiration was determined in intact HepG2 cells (original data in (van der Stel et al. 2020)). All concentrations are given as pEC_{25} in -log[M].

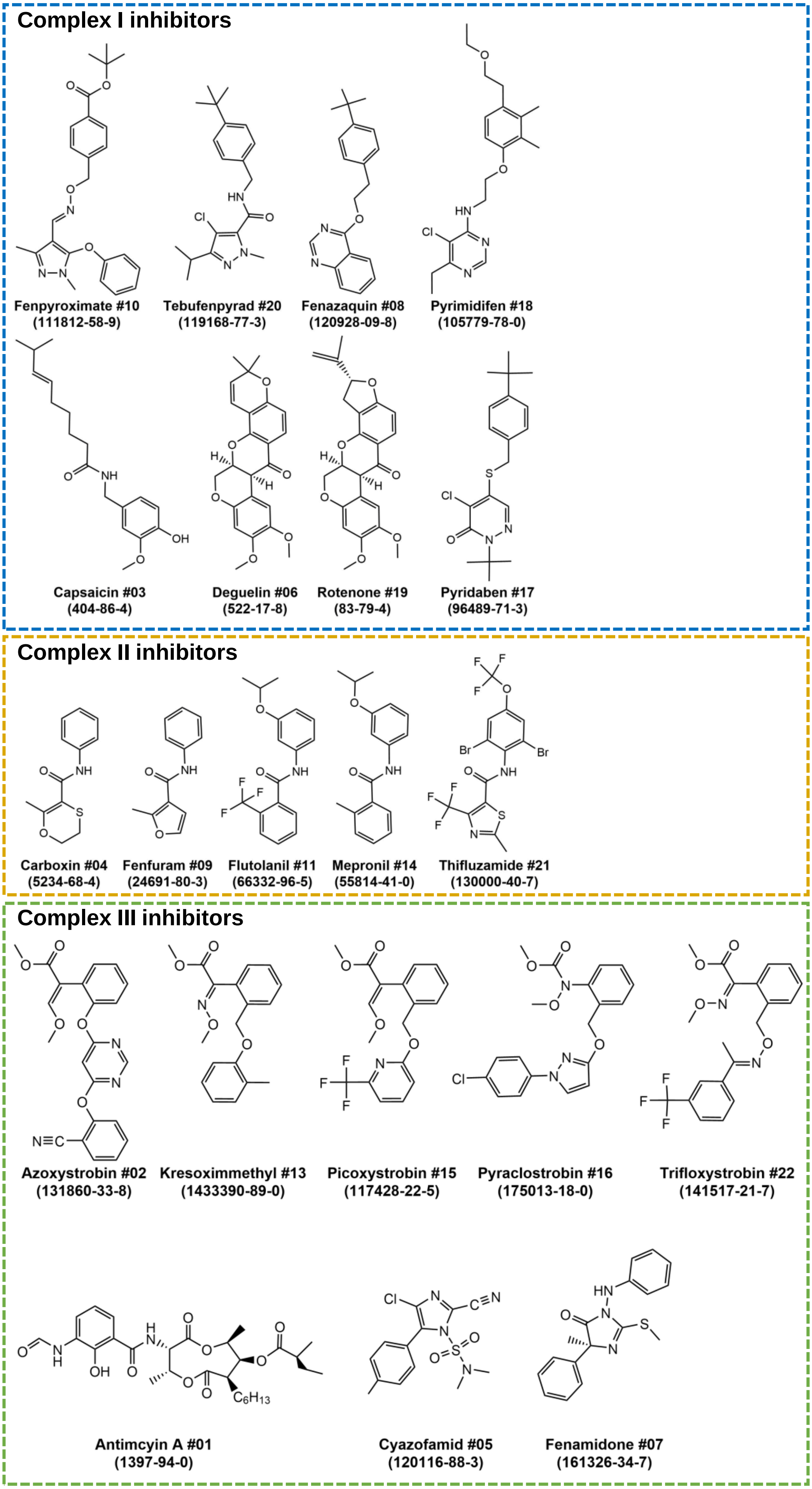
Complex I inhibitors
Fenpyroximate #10
(111812-58-9)
Tebufenpyrad #20
(119168-77-3)
Fenazaquin #08
(120928-09-8)
Pyrimidifen #18
(105779-78-0)
Capsaicin #03
(404-86-4)
Deguelin #06
(522-17-8)
Rotenone #19
(83-79-4)
Pyridaben #17
(96489-71-3)
Complex II inhibitors
Carboxin #04
(5234-68-4)
Fenfuram #09
(24691-80-3)
Flutolanil #11
(66332-96-5)
Mepronil #14
(55814-41-0)
Thifluzamide #21
(130000-40-7)
Complex III inhibitors
Azoxystrobin #02
(131860-33-8)
Kresoximmethyl #13
(1433390-89-0)
Picoxystrobin #15
(117428-22-5)
Pyraclostrobin #16
(175013-18-0)
Trifloxystrobin #22
(141517-21-7)
Antimcyin A #01
(1397-94-0)
Cyazofamid #05
(120116-88-3)
Fenamidone #07
(161326-34-7)

Suppl. Fig. 2

Suppl. Fig. 2: Chemical structures of test compounds.

Depiction of the chemical structures of all 21 compounds used in this study, grouped according to their mode of action. Some subgroups of compounds share a common chemical backbone (e.g. rotenone and deguelin, or the five strobilurins azoxy-, picooxy-, pyraclo- and trifloxystrobin as well as kresoxim-methyl). The compound identifier (ID#) corresponds to Suppl. Fig. 1. The CAS number is given below the compound name

---

Suppl. Fig. 3: Comparison of neurotoxicity after acute and chronic exposure in two models.

A) LUHMES cells were either treated according to the established NeuriTox exposure scheme (left, differentiation for two days, followed by a subsequent treatment of 24 h), or (right) treated on day 5 of differentiation for two days and retreated on d7 for another three days. For endpoint determination, cells were stained with calcein-AM and H-33342 and analyzed using high-content imaging and automated image analysis. Neurite area and viability were determined from the same pictures. **B**) Concentration-response graphs for cI (blue), cII (yellow), and cIII (green) inhibitors, ordered according to the compounds' potency within their MoA group. NeuriTox data were represented in blue (neurite area) and open black squares (viability), data of repeated dosing (RD) were presented in orange (neurite area) and open black circles (viability). Data are means ±SEM from three independent experiments. **C**) Scatter plots comparing the EC_{25} values for neurite area reduction between single (24 h) and repeated (120 h) exposure in LUHMES (partially shown in Fig. 2) and SH-SY5Y (full data set shown in Fig. 3) cells for the subset of 13 mitochondrial inhibitors (excluding capsaicin, colored according to the substances' MoA). The dotted line represents the equipotency. If the highest tested concentration did not reach effects ≥25%, >-5 or >-4 were used as surrogate; note that many compounds were inactive, thus they cluster in the >-4 or >-5 area. ND: neurite degeneration, NA: neurite area.

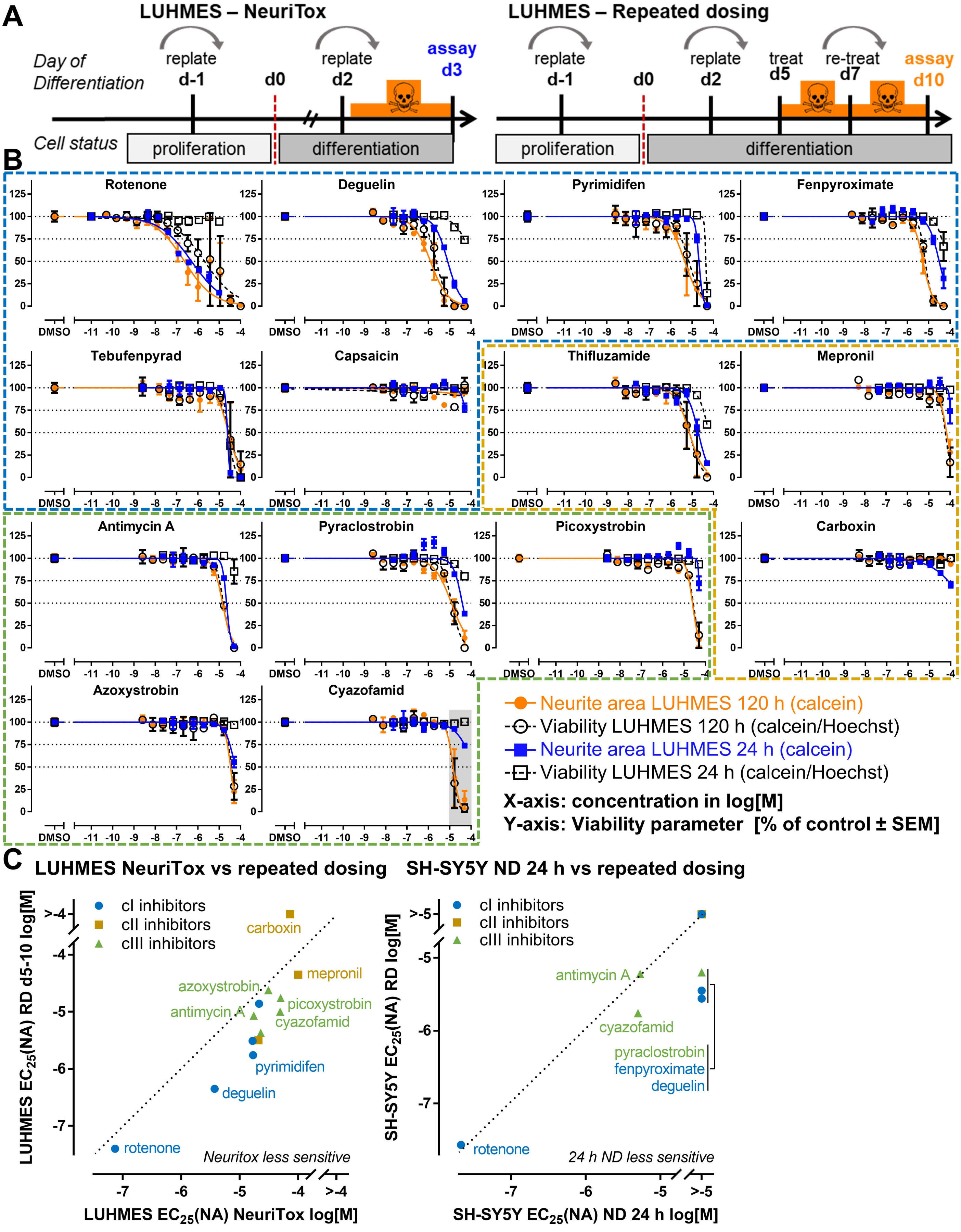
A
LUHMES – NeuriTox
LUHMES – Repeated dosing
Day of Differentiation
replate d-1
d0
replate d2
assay d3
treat d5
re-treat d7
assay d10
Cell status
proliferation
differentiation
B
Rotenone
Deguelin
Pyrimidifen
Fenpyroximate
Tebufenpyrad
Capsaicin
Thifluzamide
Mepronil
Antimycin A
Pyraclostrobin
Picoxystrobin
Carboxin
Azoxystrobin
Cyazofamid
Neurite area LUHMES 120 h (calcein)
Viability LUHMES 120 h (calcein/Hoechst)
Neurite area LUHMES 24 h (calcein)
Viability LUHMES 24 h (calcein/Hoechst)
X-axis: concentration in log[M]
Y-axis: Viability parameter [% of control ± SEM]
C
LUHMES NeuriTox vs repeated dosing
SH-SY5Y ND 24 h vs repeated dosing
cI inhibitors
cII inhibitors
cIII inhibitors
carboxin
mepronil
azoxystrobin
antimycin A
picoxystrobin
cyazofamid
pyrimidifen
deguelin
rotenone
Neuritox less sensitive
LUHMES EC25(NA) RD d5-10 log[M]
LUHMES EC25(NA) NeuriTox log[M]
pyraclostrobin
fenpyroximate
24 h ND less sensitive
SH-SY5Y EC25(NA) RD log[M]
SH-SY5Y EC25(NA) ND 24 h log[M]

Suppl. Fig. 3

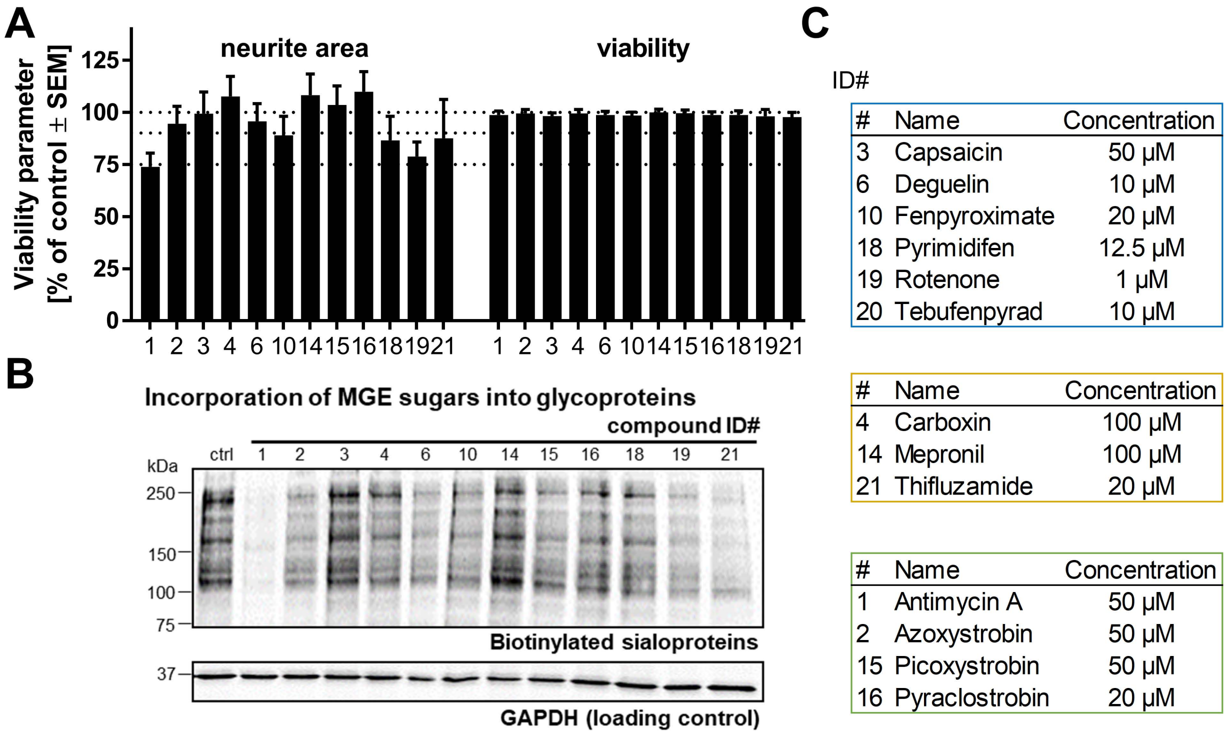

C

ID#

#	Name	Concentration
3	Capsaicin	50 µM
6	Deguelin	10 µM
10	Fenpyroximate	20 µM
18	Pyrimidifen	12.5 µM
19	Rotenone	1 µM
20	Tebufenpyrad	10 µM

#	Name	Concentration
4	Carboxin	100 µM
14	Mepronil	100 µM
21	Thifluzamide	20 µM

#	Name	Concentration
1	Antimycin A	50 µM
2	Azoxystrobin	50 µM
15	Picoxystrobin	50 µM
16	Pyraclostrobin	20 µM

Suppl. Fig. 4: Viability parameters and Western blots of the NeuroGlycoTest

A) In parallel to the assessment of neurite MGE labelling, additional parameters as neurite area and cell viability (determined by calcein-AM and H-33342 staining) were determined to demonstrate cellular intactness of samples in which glycosylation assays were performed. **B**) Representative western blots used for the quantification of Fig. 5C (sialoprotein MGE). Labelled sialic acid precursors were fed to cells. After lysis, cell proteins were separated by PAGE, and blotted (Western) on membranes. There, sialoproteins that were linked to biotin via MGE were detected by an IgG horseradish peroxidase-conjugated secondary antibody. The loading control GAPDH was detected by a primary antibody and with a horseradish peroxidase-conjugated secondary antibody. **C**) Highest non-cytotoxic concentrations of the cI-III inhibitors used in the NeuroGlycoTest, as determined from data (Fig. 2).

Regulation	Abbreviation	Gene Name	Function
up	KIF20A	Kinesin Family Member 20A	Mitotic kinesin; chromosome passenger complex (CPC)-mediated cytokinesis
up	TPX2	TPX2 Microtubule Nucleation Factor	Spindle assembly factor required for mitotic spindles and during apoptosis
up	NQO1	NAD(P)H Quinone Dehydrogenase 1	FAD-binding protein that reduces quinones to hydroquinones; major target of the Nrf-2 oxidative stress response transcription factor
up	UBE2C	Ubiquitin Conjugating Enzyme E2 C	Targeting abnormal or short-lived proteins for degradation; required for the destruction of mitotic cyclins and for cell cycle progression
up	CDKN3	Cyclin Dependent Kinase Inhibitor 3	Dephosphorylates CDK2 kinase, thus prevent the activation of CDK2 kinase and cell cycle progression
up	NEK2	NIMA Related Kinase 2	Centrosome separation and bipolar spindle formation in mitotic cells. Can lead to G1/S arrest
down	ARRDC4	Arrestin Domain-Containing Protein 4	May be involved in endocytosis of G protein-coupled receptors and glucose uptake
down	CALR	Calreticulin	Major Ca(2+)-binding (storage) protein in the lumen of the endoplasmic reticulum and nucleus. Regulator of gene expression
down	KIF5C	Kinesin Family Member 5C	Neuro-specific. Anterograde transport of cargo (mRNA, organelles) within the central nervous system
down	MMP2	Matrix Metalloproteinase-2	Cleavage of components of the extracellular matrix and molecules involved in signal transduction
down	TXNIP	Thioredoxin Interacting Protein	Inhibits the antioxidative function of thioredoxin; inhibits the proteasomal degradation of DDIT4
down	POU4F2	POU Domain, Class 4, Transcription Factor 2	Transcription factor involved in the development and differentiation of neuronal cells
down	TNC	Tenascin C or Neuronectin	Extracellular matrix protein with multiple EGF-like and fibronectin type-III domains. Guidance of migrating neurons as well as axons during development and neuronal regeneration

Suppl. Fig. 5: Background information on the genes found to be regulated in LUHMES cells by two non-toxic deguelin concentrations

Tabular overview about the genes that were identified to be regulated in LUHMES cells treated with deguelin at a concentration causing 10% cell death (12 µM), and a quarter of it (3 µM). The first six genes were upregulated due to the treatment, the lower seven genes were found to be downregulated. Information on the function of the genes was retrieved from www.genecards.org.

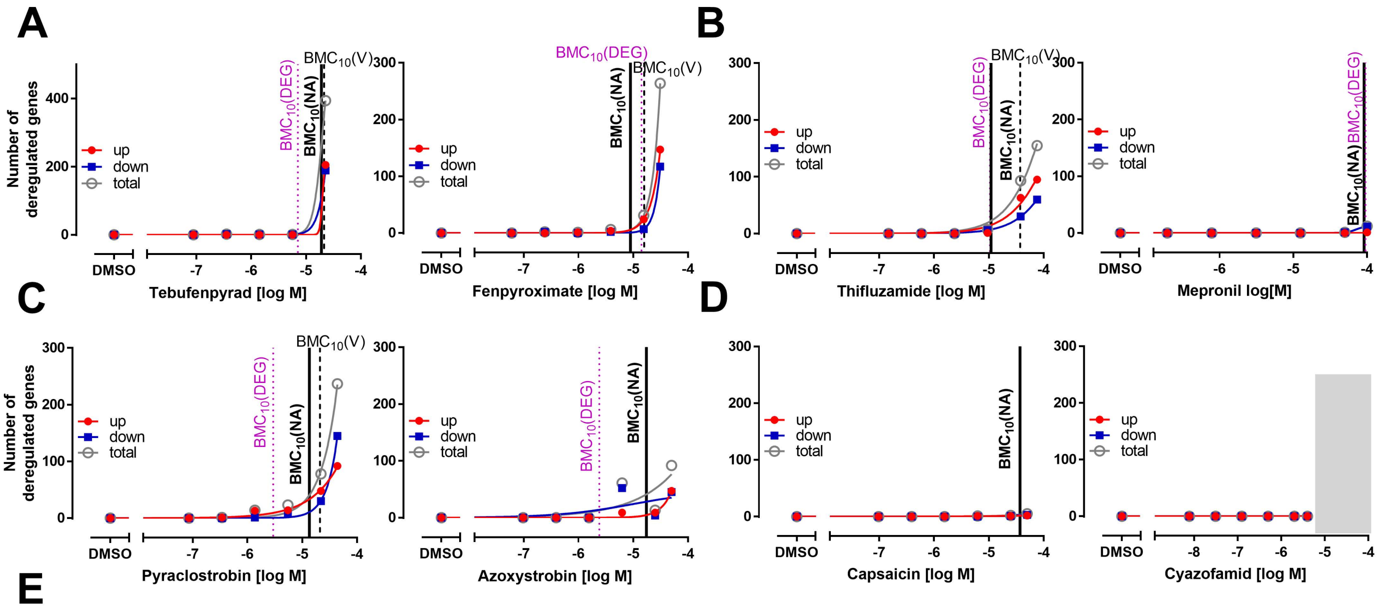

E

ID#	Compound	Target	BMC_{10} [µM] NA	Genes	More sensitive Endpoint
06	Deguelin	cI	1.8	3.0	Equal
19	Rotenone	cI	0.01	0.04	Neurites
10	Fenpyroximate	cI	8.9	14	Equal
18	Pyrimidifen	cI	13	6.3	Genes
20	Tebufenpyrad	cI	19	7.1	Genes
04	Carboxin	cII	13	>100	Neurites
14	Mepronil	cII	91	95	Equal
21	Thifluzamide	cII	11	10.5	Equal
01	Antimycin A	cIII	13	18.6	Equal
02	Azoxystrobin	cIII	17	2.4	Genes
15	Picoxystrobin	cIII	13	6.0	Genes
16	Pyraclostrobin	cIII	13	3.0	Genes
03	Capsaicin	other	37	>100	Neurites
05	Cyazofamid	other	n.d.	n.d.	n.d.

Note: to better define the concentrations to be tested in the TempoSeq assay, we repeated the NeuriTox test for these 14 compounds again with closer spacing of the concentrations. Of course, this revealed *slightly* different EC_{10} concentrations than in Fig. 3. Issues can be found for rotenone, where no $EC_{10}V$ could be defined from the data set in Fig. 3, but in the retesting V dropped to 90% of control and was there stable, thus an $EC_{10}V$ was defined. All other assays except from Temposeq were done before the retesting/Biospyder and thus orient on the EC values derived from Fig. 3.

Suppl. Fig. 6

Suppl. Fig. 6: Concentration-response analysis of the number of differentially expressed genes

LUHMES cells were differentiated for 48 h and subsequently treated for 24 h with cI (A), cII (B), cIII (C) inhibitors or substances with another mode of action (D). Analysis of the transcriptome (mRNA expression) was performed using Biospyder's TempO-Seq technique. Tested concentrations were anchored on the viability impairment identified in the NeuriTox test (EC10V) and are as follows: twice the EC10(V), EC10(V), and 1/4, 1/16, 1/32, 1/64 of the EC10(V). The test substances mepronil, carboxin, picoxystrobin, azoxystrobin, capsaicin, cyazofamid reduced viability by only <10%, thus no EC10(V) could be determined. Therefore, the highest possible concentration (100 or 50 µM) was used as starting point and subsequently diluted once 1:2, then in 1:4 steps. For pyrimidifen and tebufenpyrad, the amount of RNA retrieved from the 2-times EC10(V) samples was not sufficient for gene expression analysis due to extensive cell lysis. The green vertical line indicates the BMC50 of respiration inhibition of the specific respiratory chain complex assessed in LUHMES (assessed for rotenone, deguelin, antimycin A).
The solid vertical black line indicates the EC_{10} of neurite outgrowth impairment (BMC_{10}(NA)), the dashed vertical black line indicates the EC_{10} of viability impairment (BMC_{10}(V)). The dotted pink line indicates the BMC_{10} of gene regulation (BMC_{10}(DEG)). DEG were defined by a fold change >1.5 and padj >0.05. For the calculation of the BMC_{10}(DEG), see Fig. 6. **E)** Synoptic overview about the ratio of BMC10 values of neurite outgrowth (NA) and gene expression dysregulation (genes). n.d.: not determined due to low effect. Differences that were < 2-fold were considered to be non-significant (=equal).

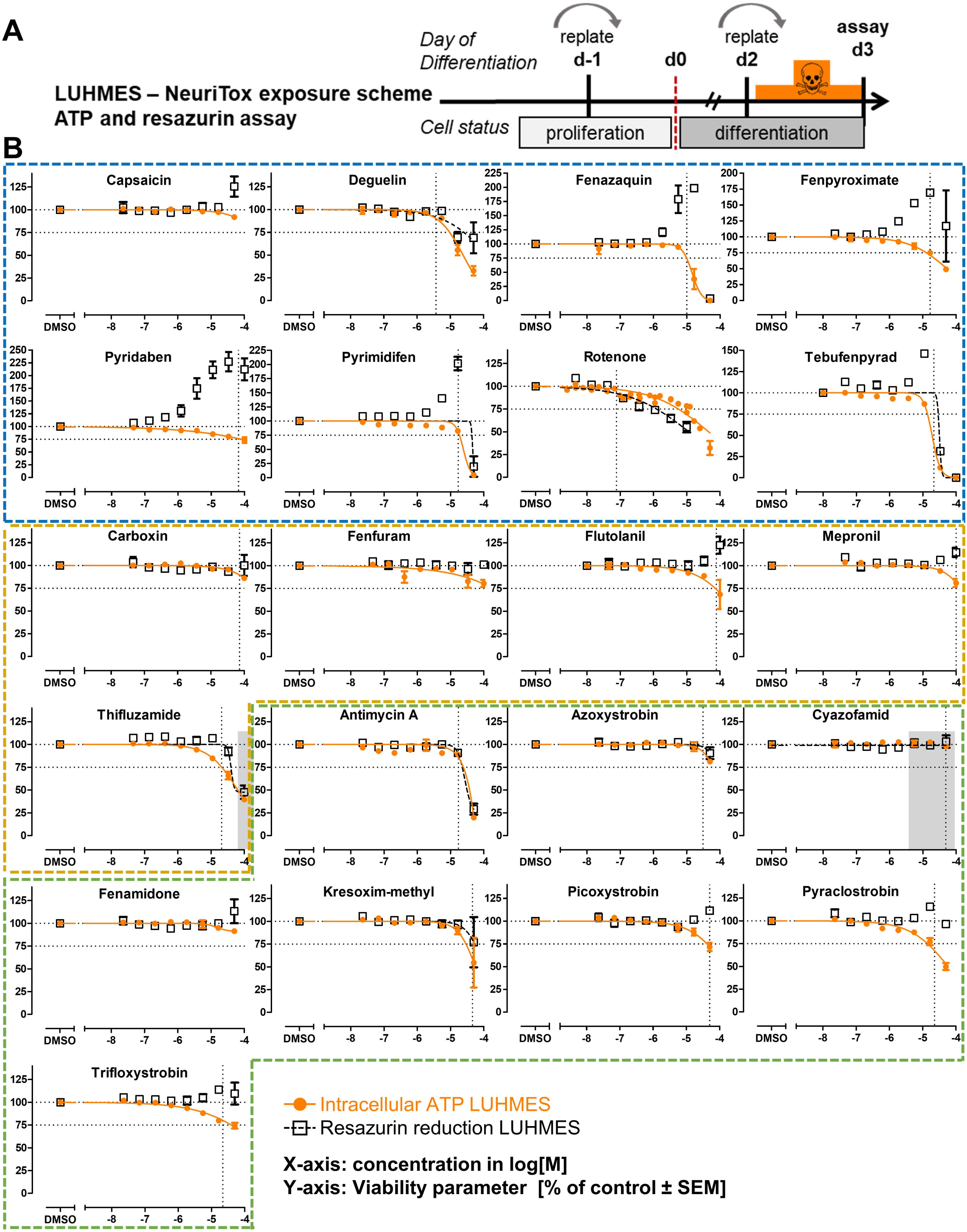
A
Day of Differentiation
replate d-1
d0
replate d2
assay d3
LUHMES – NeuriTox exposure scheme
ATP and resazurin assay
Cell status
proliferation
differentiation
B
Capsaicin
Deguelin
Fenazaquin
Fenpyroximate
Pyridaben
Pyrimidifen
Rotenone
Tebufenpyrad
Carboxin
Fenfuram
Flutolanil
Mepronil
Thifluzamide
Antimycin A
Azoxystrobin
Cyazofamid
Fenamidone
Kresoxim-methyl
Picoxystrobin
Pyraclostrobin
Trifloxystrobin
DMSO
Intracellular ATP LUHMES
Resazurin reduction LUHMES
X-axis: concentration in log[M]
Y-axis: Viability parameter [% of control ± SEM]

Suppl. Fig. 7

Suppl. Fig. 7: Assessment of intracellular ATP levels and resazurin reduction in the NeuriTox test

A) The same assay plates that were used for the LUHMES NeuriTox test (endpoint determination d3 of differentiation after 24 h treatment) were analyzed for their intracellular ATP content (orange) and resazurin reduction (black). **B**) Concentration-response graphs for cI (blue), cII (yellow), and cIII (green) inhibitors, ordered according to the compounds' potency within their MoA group. The vertical lines indicate the EC25 of the NeuriTox test (Fig. 2). Data are means ±SEM from three independent experiments. No curve fits were performed for resazurin data, as the complex forms would require non-monotonic modelling. For the ATP-data, the dotted line indicates the threshold of a 25% drop. Data corresponding exactly to such a drop (EC25ATP) in the curve fit model were obtained and are shown in figure 3.

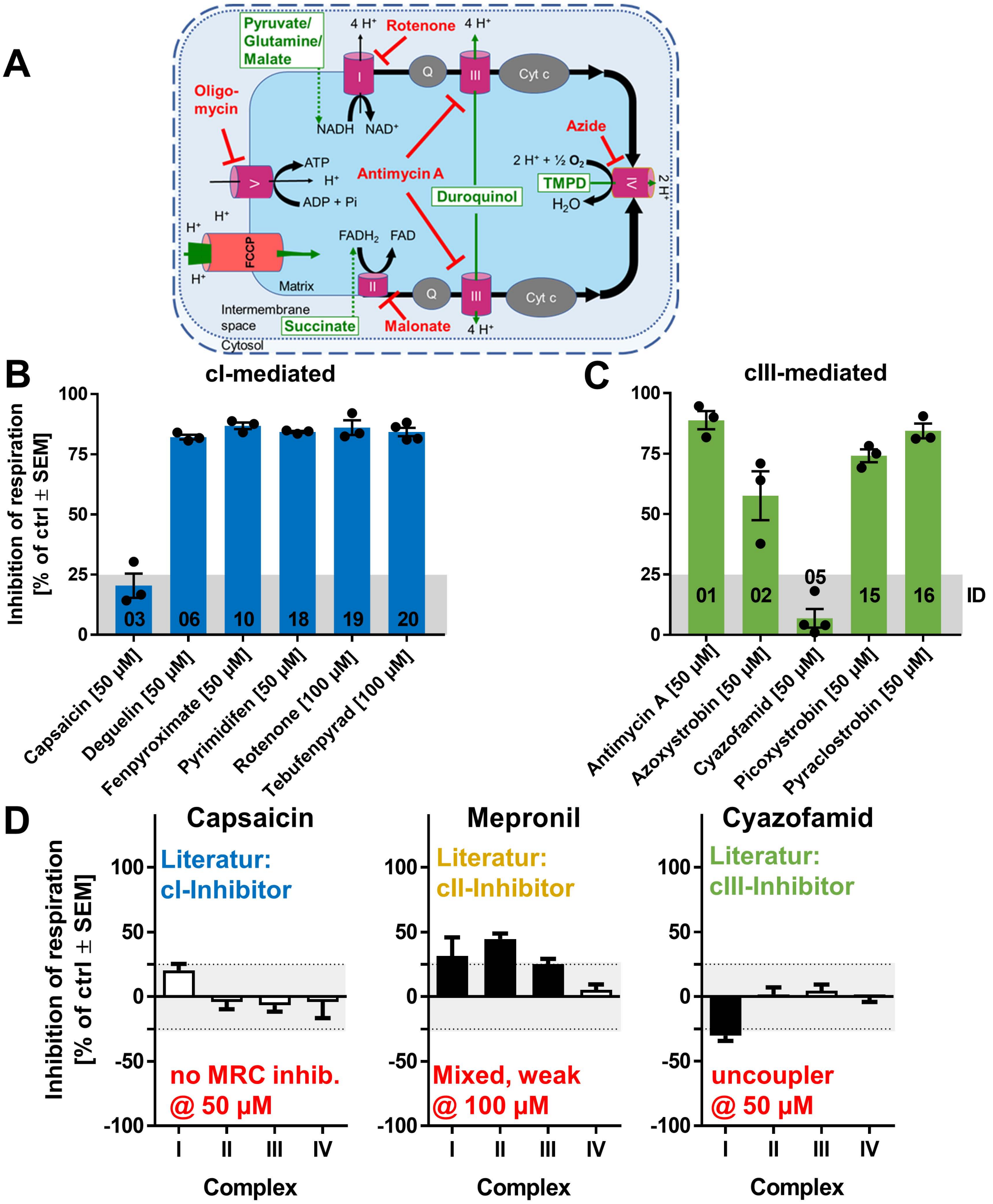

Suppl. Fig. 8: Schematic representation of the mitochondrial complex activity assay and data for cI and cIII inhibition.

A) Cells were permeabilized to gain direct access to their functional mitochondria without purification steps. Each individual complex of the respiratory chain can be fueled by specific substrates (green), or inhibited by specific inhibitors (red). Changes in complex activity can be monitored by quantification of cIV-mediated oxygen consumption. **B&C**) Impairment of specific cI activity by cI inhibitors and specific cIII activity by cIII inhibitors (tested at their highest possible (i.e. 1:1000 of stock) concentration) was assessed using proliferating permeabilized LUHMES cells as described in A). The numbers at the bottom of the bars indicate the compound ID. Bars represent means ±SEM, and each point represents the result of an independent experiment. The gray area is the noise band of control+25% as defined in (Delp et al. 2019), i.e. inhibition within this range is regarded to be insignificant. Data not shown: cII-IV activity was not affected by cI inhibitors; cI, cIII and cIV activity was not affected by cII inhibitors; and cIV activity was not affected by cIII inhibitors. **D**) Full spectrum of specific cI-cIV activities of permeabilized LUHMES cells treated with 50 µM capsaicin, 100 µM mepronil or 50 µM cyazofamid, i.e. the three of the 21 substances that were found in our assay to behave different from our initial expectations (based on literature). For mepronil, a specific cII inhibition was not detectable, but rather a weak inhibition of cI-III activity. This may be due to a cIII effect (resulting in apparent cI and cII inhibition), or an unspecific inhibition of the respiratory chain. Cyazofamid did not alter cII-IV activity significantly, but resulted in an apparent increased cI activity. This pattern is indicative for uncoupling compounds, as explained in (Delp et al. 2019) Data are means ±SEM from three-four independent experiments.

Target	Triggering of KE4 (AO) @ 50 µM Neurite toxicity	ID	Substance	KE4 agrees with			
				KE3 proteasomal	KE2 MMP	KE1 total respiration	KE1 complex assay
cI inhibitors	+	19	Rotenone	✓	✓	✓	✓
	+	06	Deguelin	✓	✓	✓	✓
	+	18	Pyrimidifen	✓	✓	✓	✓
	+	10	Fenpyroximate	✓	✓	✓	✓
	+	20	Tebufenpyrad	n.d.	✓	✓	✓
cII inhib.	O	04	Carboxin	✓*	n.c.	✓*#	- #
	O	14	Mepronil	✓*	n.c.	✓*#	- §
	+	21	Thifluzamide	✓	n.d.	n.d.	✓#
cIII inhib.	+	01	Antimycin A	✓	✓	✓	✓
	+	02	Azoxystrobin	n.c.	✓	✓	✓
	+	15	Picoxystrobin	n.d.	✓	✓	✓
	+	16	Pyraclostrobin	n.c.	✓	✓	✓
	O	05	Cyazofamid	n.d.	n.c.	n.d.	✓

Suppl. Fig. 9: Overview of AOP-consistent triggering of key events.

Synoptic overview of KE assay concordance. Binary classification for consistent triggering of key events (KE), anchored on the results of the KE4(AO) assay NeuriTox test for neurite toxicity at 50 µM. If the substance caused ≥25% inhibitory effects, it was labeled with "+", if the inhibitory effects were <25% at 50 µM, it was labeled "O". When the KE1-3 assays showed ≥25% effect, and the KE4 assay also showed ≥25% effect, the respective KE1-3 box was labeled with "✓" (i.e. coherent activation). When the KE1-3 assays showed <25% effect, and the KE4 assay also showed <25% effect, the respective KE1-3 box was labeled with "✓*" (i.e. coherent non-activation). When the KE4 assay showed ≥25% effect, but the KE1-3 assay did not (or vice versa), the respective box was labeled with "n.c." (i.e. incoherent (non-)activation). Note: data on respirometric inhibition were obtained from LUHMES cell cultures. Cyazofamid was insoluble at 50 µM and rather behaved like an uncoupler than a cIII inhibitor. Thus it was listed separately from cIII inhibitors. §: mepronil behaved like a weak mixed inhibitor at 100 µM (cII inhibition of 45%). #: thifluzamide was tested to be a strong and specific cII inhibitor at 100 µM (cII inhibition of 84%), carboxin reduced cellular respiration by 33% at 100 µM, mepronil reduced cellular respiration by 30% at 100 µM. N.d.: not determined.

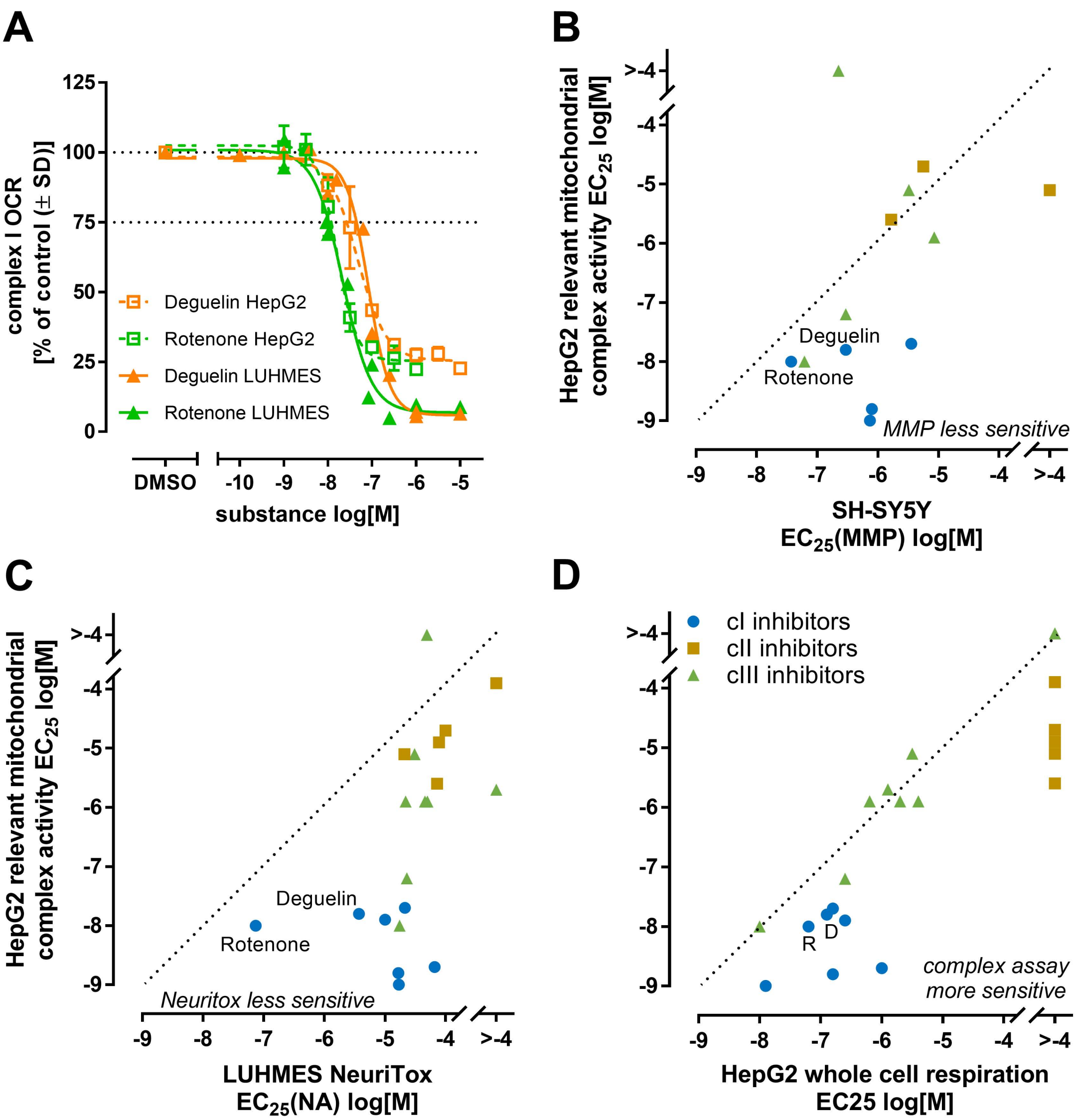

Suppl. Fig. 10: Scatter plots comparing the sensitivity of different KE assays and their derivatives with each other.

Data for HepG2 cells (van der Stel et al. 2020) were used here for comparison, as LUHMES data are available only for fixed concentrations. **A**) The basal cI-mediated OCR assay was similarly sensitive in LUHMES (NeuriTox) and HepG2 cells for rotenone (green full triangles and empty squares, respectively) and deguelin (orange full triangles and empty squares, respectively). **B**) The mitochondrial complex activity assay is more sensitive than the NeuriTox assay. Based on all 21 compounds, excluding capsaicin. **C**) For many cI inhibitors (blue), the SH-SY5Y based MMP assay was less sensitive compared to the permeabilized HepG2 cells. For cII (yellow) and cIII (green) inhibitors, similar sensitivities were detected. Based on the subset of 14 compounds, excluding capsaicin. **D**) Comparing the respiration impairment of intact and permeabilized HepG2 cells shows that the assay of individual complexes in permeabilized cells is more sensitive for cI (blue) and cII inhibitors (yellow), but not for cIII inhibitors (green). Data are shown for 20 compounds, i.e. without capsaicin. Data are means ±SEM from three independent experiments, unless mentioned otherwise.

MoA	EC25 KE4 (AO) Neurite toxicity	ID	Substance	EC25 values		Ratios of EC25	
				KE1 total respiration	KE1 complex assay	KE4/KE1 total respiration	KE4/KE1 complex assay
cI i.	5	08	Fenazaquin	6.6	7.9	39.8	800.0
	4.2	17	Pyridaben	6	8.7	63	32000
cII i.	<4	09	Fenfuram	<5	<4	n.a.	n.a.
	4.1	11	Flutolanil	<5	4.9	n.a.	6
cIII i.	<4.3	07	Fenamidone	5.9	5.7	>40	>25
	4.3	13	Kresoxim-methyl	5.4	5.9	12.6	39.8
	4.7	22	Trifloxistrobin	5.7	5.9	10	15.8
	<4.3	03	Capsaicin	<5	<4	n.a.	n.a.

Suppl. Fig. 11: Comparison of the sensitivities of different assays along the AOP.

Synoptic overview of the EC25 concentrations (in –log[M]) of KE1,2 and 4 assays along the AOP for the eight toxicants which have not been characterized in Fig 9. Anchoring point is the EC25 concentration for neurite outgrowth impairment of the NeuriTox test for KE4 (in vitro proxy for the AO). EC25 concentrations of other KE-assays were displayed as ratio (e.g. KE4/KE1) and colored in faint red if >3, in red if >10 and dark red if >100 (i.e. upstream KE1-3 assay was more sensitive than KE4) and white for ratios between 3 and 1/3. Note: data on respirometric inhibition were retrieved from HepG2 based assays (van der Stel et al. 2020); n.a.: ratio could not be determined due to low effects; n.t.: not tested. Capsaicin was in this study not confirmed to be a cI inhibitor.

Suppl. Data: Differential gene expression analysis results for 14 mitochondrial inhibitors

Transcriptomics data of LUHMES exposure to five or six different concentrations for each of the 14 test compounds was analyzed using the deseq2 R package. For each treatment, results are displayed on a separate spreadsheet and contain the associated metadata (Gene_ID, Entrez_ID, Treatment, Concentration [µM]) and statistical parameters of the Wald test (log2FoldChange, lfcSE, stat, pvalue and padj). The log2 fold-change (log2FoldChange) values represent the means of 3 biological replicates. The standard error (lfcSE) is also given on the log2 scale. To adjust for multiple comparisons, the Benjamini-Hochberg method was applied, resulting in adjusted P-values (padj). The data are found in a separate Excel file with a separate worksheet for each component.
